# Human colonic EVs induce murine enteric neuroplasticity via the lncRNA GAS5/miR-23/NMDA NR2B axis

**DOI:** 10.1172/jci.insight.178631

**Published:** 2025-03-10

**Authors:** QiQi Zhou, Liuqing Yang, Zachary T. Verne, Benjamin B. Zhang, Jeremy Z. Fields, Amber T. Thacker, G. Nicholas Verne

**Affiliations:** 1 College of Medicine, University of Tennessee Health Science Center, Memphis, Tennessee, USA.; 2 Lt. Col. Luke Weathers, Jr. VA Medical Center, Research Service, Memphis, Tennessee, USA.

**Keywords:** Gastroenterology, Epigenetics, Noncoding RNAs

## Abstract

Postinfectious, diarrhea-predominant, irritable bowel syndrome (PI-IBS-D) is difficult to treat owing to its unknown pathophysiology. Extracellular vesicles (EVs) derived from human colon tissue and long noncoding RNAs (lncRNAs), such as growth arrest–specific 5 (GAS5), may play key roles in the pathophysiology of PI-IBS-D. To determine whether altered colonic EV lncRNA signaling leads to gastrointestinal dysfunction and heightened visceral nociception in patients with PI-IBS-D via the GAS5/miR-23ab/NMDA NR2B axis, we conducted translational studies, including those on (a) the role of colonic EV lncRNAs in patients with PI-IBS-D, human colonoids, and PI-IBS-D tissues; (b) i.p. injection of colonic EVs from patients with PI-IBS-D into Rab27a/b^–/–^ mice (P-EV mice) to investigate whether colonic EVs drive visceral hypersensitivity in vivo via the GAS5/miR-23ab/NMDA NR2B axis; and (c) treatment of mice with oligo-miR-23 precursors and anti-GAS5 Vivo-Morpholinos for GAS5/miR-23ab/NMDA NR2B axis mechanisms. Colonic EVs from patients with PI-IBS-D, but not from control participants, demonstrated reduced miR-23a/b expression caused by enhanced GAS5 expression, which drives increased NR2B expression. Intraperitoneal injection of anti–GAS5-Vivo-Morpholino into P-EV mice increased miR-23 levels and decreased NR2B expression and VMR to CD. EVs are internal messengers that alter gastrointestinal function and increase visceral nociception in patients with PI-IBS-D. Strategies to deliver EVs to modulate GAS5/miR-23ab/NMDA NR2B axis signaling may lead to new and innovative treatments for patients with PI-IBS-D.

## Abstract 2

Some patients who develop food poisioning go on to develop gastrointestinal symptoms after their infection of the gut has cleared. The current proposal will investigate the underlying causes of these chronic symptoms by examining biological factors (neurotransmitters and genes) that modulate the changes in both humans and mice. Knowing the biological basis of these gastrointestinal symptoms should allow for new therapeutic approaches and greatly facilitate the development of better drugs for treating these patients.

## Introduction

Postinfectious, diarrhea-predominant, irritable bowel syndrome (PI-IBS-D) is a gastrointestinal disorder that often develops following enteric infections and is characterized by abdominal pain, diarrhea, and urgency (1). The mechanisms underlying the persistent gastrointestinal symptoms in patients with PI-IBS-D are unknown, but interactions between enteric pathogens and the intestinal epithelium may be involved, leading to alterations in gastrointestinal function, motility, and visceral nociception (2–12). The unclear pathophysiology of PI-IBS-D has resulted in pharmacologic therapies that are limited in their effectiveness (13, 14).

Recently, extracellular vesicles (EVs), including exosomes and microvesicles, have received considerable interest because of their role in intercellular communications and their potential clinical applications (15–18). EVs can deliver molecules and signals from the gastrointestinal tract to cells in other organ systems, such as the nervous system (19, 20). EV-associated long noncoding RNAs (lncRNAs) and circular RNAs participate in many hallmarks of GI disorders. These noncoding RNA cargos can be exploited as biomarkers for various human GI diseases. EVs can be used to deliver therapeutic RNAs (lncRNAs and miRNAs) for gene therapy (18).

In this study, we hypothesized that human colonic EVs directly drive the development of chronic gastrointestinal symptoms in patients with PI-IBS-D, following enteric infections. The goal of this study was to identify lncRNA/miRNA expression, signaling, and targets in human colonic EVs from patients with PI-IBS-D. Second, we determined whether lncRNA growth arrest–specific 5 (GAS5) competes with miR-23a/b for binding sites and leads to decreased miR-23, subsequently increasing NMDA NR2B expression, as well as postinflammatory and neuropathic pain signaling. Finally, given the potential involvement of EVs in the development of PI-IBS-D, we sought to determine whether EVs act as internal messengers that alter gastrointestinal visceral nociception and function and contribute to the pathophysiology of PI-IBS-D.

## Results

First, to enable us to extract EVs, we recruited 45 participants, including 27 consecutive patients with PI-IBS-D diagnosed after *Campylobacter* infection (*n* = 16), after *Salmonella* infection (*n* = 6), and after Shiga toxin–producing *E*. *coli* infection (*n* = 5). These 27 patients with PI-IBS-D included 19 female and 8 male patients (mean age, 29.2 ± 8.5 years) from the Gastroenterology Clinics at Tulane University and the University of Tennessee Health Science Center and 18 age- and sex-matched participants in the control population (12 female and 6 male patients; mean age, 30.4 ± 6.8 years). All patients with PI-IBS-D had a previous enteric infection documented in their medical records at least 1 year earlier and met the Rome IV Criteria for PI-IBS-D (21). All participants underwent both a hydrogen breath test (to exclude bacterial overgrowth) and a tissue transglutaminase immunoglobulin A antibody titer (to exclude celiac sprue). Random colon biopsies did not reveal any microscopic evidence of intestinal inflammation or morphological changes in villi.

### Colonic EVs and miR-23a/b and NMDA NR2B expression in patients with PI-IBS-D

#### Colon EV-miR-23a/b in patients with PI-IBS-D.

**Figure 1:**
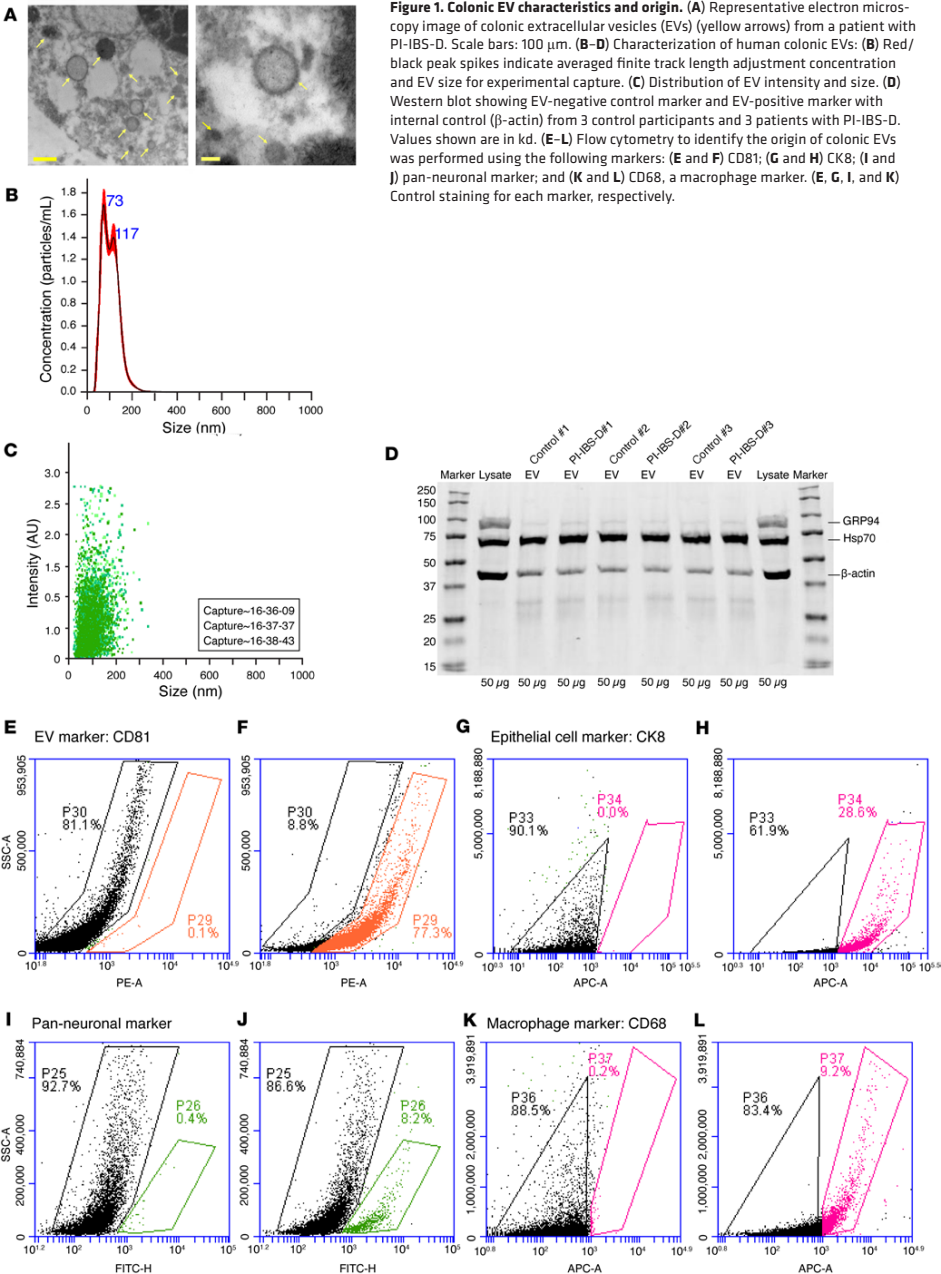
Colonic EV characteristics and origin. (**A**) Representative electron microscopy image of colonic extracellular vesicles (EVs) (yellow arrows) from a patient with PI-IBS-D. Scale bars: 100 μm. (**B**–**D**) Characterization of human colonic EVs: (**B**) Red/black peak spikes indicate averaged finite track length adjustment concentration and EV size for experimental capture. (**C**) Distribution of EV intensity and size. (**D**) Western blot showing EV-negative control marker and EV-positive marker with internal control (β-actin) from 3 control participants and 3 patients with PI-IBS-D. Values shown are in kd. (**E**–**L**) Flow cytometry to identify the origin of colonic EVs was performed using the following markers: (**E** and **F**) CD81; (**G** and **H**) CK8; (**I** and **J**) pan-neuronal marker; and (**K** and **L**) CD68, a macrophage marker. (**E**, **G**, **I**, and **K**) Control staining for each marker, respectively.

To determine whether EVs act as internal messengers that alter gastrointestinal function and visceral nociception, and contribute to the pathophysiology of PI-IBS-D, we extracted EVs from colon biopsies of patients with PI-IBS-D and control participants. A representative electron microscopy image of colonic EVs from a patient with PI-IBS-D is shown in Figure 1A. Next, nanoparticle tracking analysis (NanoSight, NS300) was performed on colonic EVs to determine the colonic EVs size and distribution. The double red/black peak spike indicates the averaged finite track length adjustment concentration and EV size for experimental capture (Figure 1B). Green scatter plot indicates the distribution of the EV intensity and size (Figure 1C). Figure 1D illustrates a Western blot for EV purity, the EV-negative control marker (GRP94 antibody), and the EV-positive marker (Hsp70 antibody), with an internal control (β-actin). To determine the cellular origin of colonic EVs, flow cytometry was performed to examine the EV distribution in patients with PI-IBS-D. Approximately 77.3% of the isolated EVs were positively identified using the CD81 EV marker (Figure 1, E and F). In addition, 28.6% of EVs were from colonic epithelial cells (CK8) (Figure 1, G and H), 8.2% were from colonic neurons (pan-neuron) (Figure 1, I and J), and 9.2% were from macrophages (CD68) (Figure 1, K and L).

**Figure 2:**
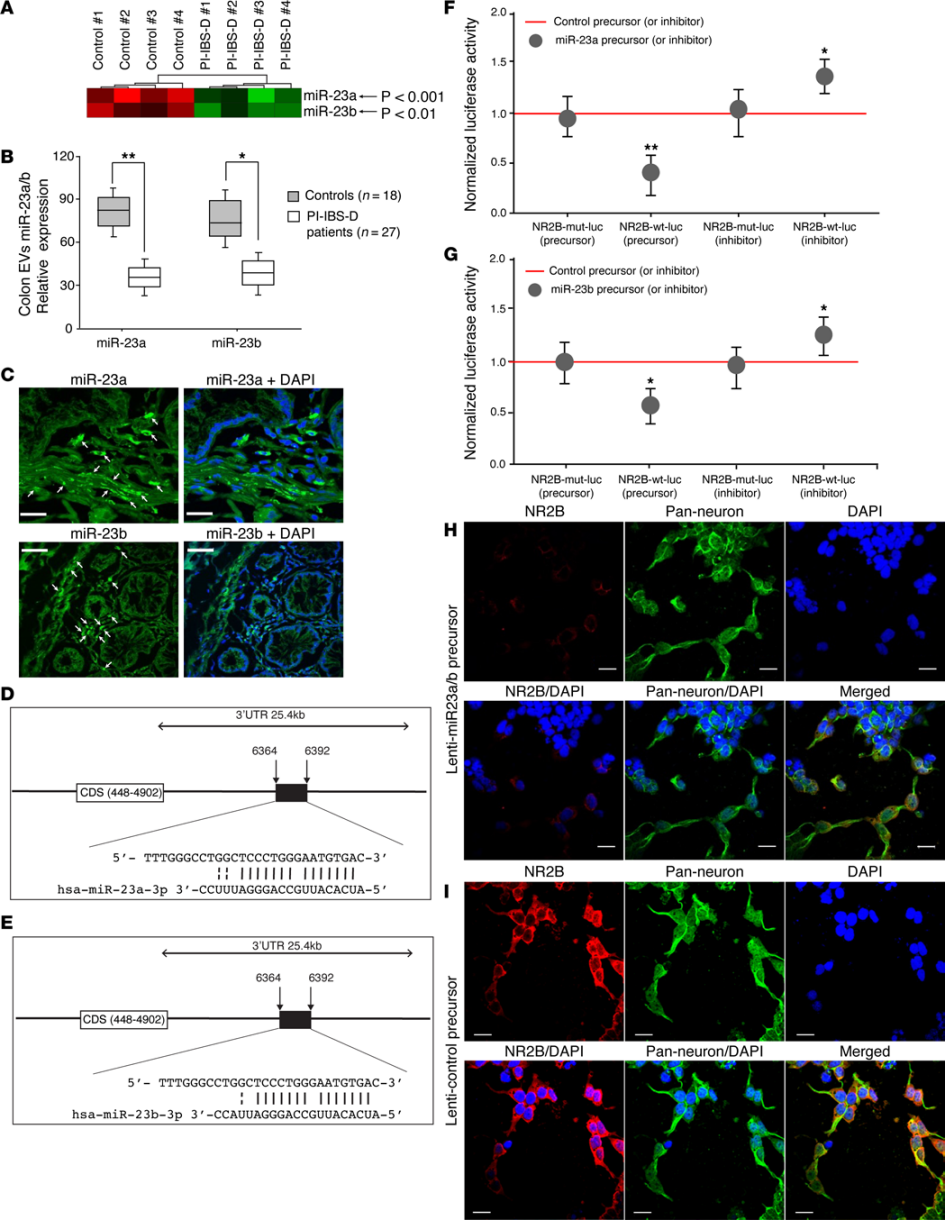
Colonic EV miR-23a/b and NMDA NR2B expression in patients with PI-IBS-D and control participants. (**A**) miRNA microarray of colonic EVs from patients with PI-IBS-D and control participants. (**B**) PCR analysis of colon EVs. (**C**) FISH analysis of miR-23a/b expression in human colon tissues. Localization of miR-23a (top) and miR-23b (bottom) expression in normal human colon. Scale bars: 50 μm. (**D** and **E**) Highly conserved miR-23a/b binding site at the NMDA NR2B mRNA 3’-UTR. (**F** and **G**) Relative luciferase reporter activity; mi-23a (**F**) and mi-23b (**G**). (**H** and **I**) Confocal double-labeling immunofluorescence staining of human neurons: (**H**) lenti-miR-23a/b precursor transfection versus (**I**) lenti-control precursor. Scale bars: 20 μm. *P = 0.05, **P = 0.01 by unpaired *t* test.

Recent evidence has demonstrated that epigenetic mechanisms such as noncoding RNA may have a pathophysiological role in the development of IBS (22). To determine whether colon EVs contain specific signaling molecules such as noncoding RNA, we performed miRNA profiling of colonic EVs from patients with PI-IBS-D (*n* = 4) and control participants (*n* = 4) using miRNA microarrays. Of the 460 miRNAs identified, cluster analysis revealed that both miR-23a and miR-23b expression was significantly downregulated (*P* = 0.001 and *P* = 0.01, respectively; Figure 2A) in colonic EVs from patients with PI-IBS-D compared with control participants. Decreased colon EV miR-23a/b expression in patients with PI-IBS-D was further confirmed by TaqMan microRNA PCR (Figure 2B) in patients with PI-IBS-D (*n* = 27) compared with control participants (*n* = 18). Downregulated miR-23a/b expression was also observed in colon biopsies from patients with PI-IBS-D (Supplemental Figure 1A; supplemental material available online with this article; https://doi.org/10.1172/jci.insight.178631DS1). miR-155, which was significantly increased in PI-IBS-D EVs and colon biopsies, was used as the positive control (Supplemental Figure 1B). Colocalization of miR-23a and miR-23b was observed in these colon biopsies using FISH analysis in 1 patient (Figure 2C). Pearson’s correlation analysis revealed a strong inverse correlation between colonic EV miR-23a and miR-23b expression and Visual Analogue Scale (VAS) abdominal pain scores in patients with PI-IBS-D (Table 1). Similarly, colonic expression of miR-23a and miR-23b in patients with PI-IBS-D also exhibited a negative (inverse) correlation with VAS abdominal pain scores.

A total of 27 patients with PI-IBS-D were included in the current study including in study; Supplemental Figure 2 shows miR-23a/b expression in subgroups of patients with IBS-D with a history of *Campylobacter* infection (*n* = 16), *Salmonella* infection (*n* = 6), and Shiga toxin–producing *E*. *coli* infection (*n* = 5). There was no significant difference in miR-23a/b expression between groups (Supplemental Figure 2).

#### Target gene of miR-23a/b — NR2B in patients with PI-IBS-D.

To further understand the link among miR-23a/b, colonic EVs, and abdominal pain, we identified molecular targets of miR-23a/b. The Sanger miRBase database target prediction program revealed a highly conserved miR-23a/b binding site in the 3′-UTR of NMDA mRNA. This binding site encodes coding sequences 488-4,902, a well-characterized NMDA glutamate receptor subtype (Figure 2, D and E). There was perfect complementarity between miR-23a/b and the 3′-UTR of Grin2B (the gene that encodes NMDA receptor subtype 2B) over the first 7 nucleotides (including the core seed, bases 2−7), which is consistent with the region serving as a bona fide miR-23a/b-binding site (Figure 2, D and E).

The relative luciferase reporter activity of HEK293T cells cotransfected with miR-23a/b precursors and the psiCHECK-2-Grin2B-WT vector was significantly lower than that of HEK293Tcells transfected with the psiCHECK-2-Grin2B-MUT (mutant) vector. In contrast, HEK293T cells transfected with psiCHECK-2-Grin2B-WT and miR-23a/b inhibitors displayed increased luciferase activity compared with those transfected with psiCHECK-2-Grin2B-MUT (Figure 2, F and G). A highly conserved binding site for miR-23a/b in the NMDA mRNA 3’-UTR was also observed in mouse sequences was verified in mouse YAMC cells (Supplemental Figure 3). Cell cultures were performed to test the mechanistic relationship between NR2B and miR-23a/b. Confocal double-labeling immunofluorescence staining of human neurons revealed that NMDA NR2B expression was reduced after lenti-miR-23a/b precursor transfection (Figure 2H) versus that with lenti-control precursor (Figure 2I). Because miRNAs usually have multiple targets, luciferase assays for CXCR4 and CXCL12, as targets of miR-23a/b, were also performed (Supplemental Figures 4 and 5), and the details are provided in the legends for the supplemental figures.

### NMDA NR2B expression in colonic EVs, colon tissue, and cells from patients with PI-IBS-D

**Figure 3:**
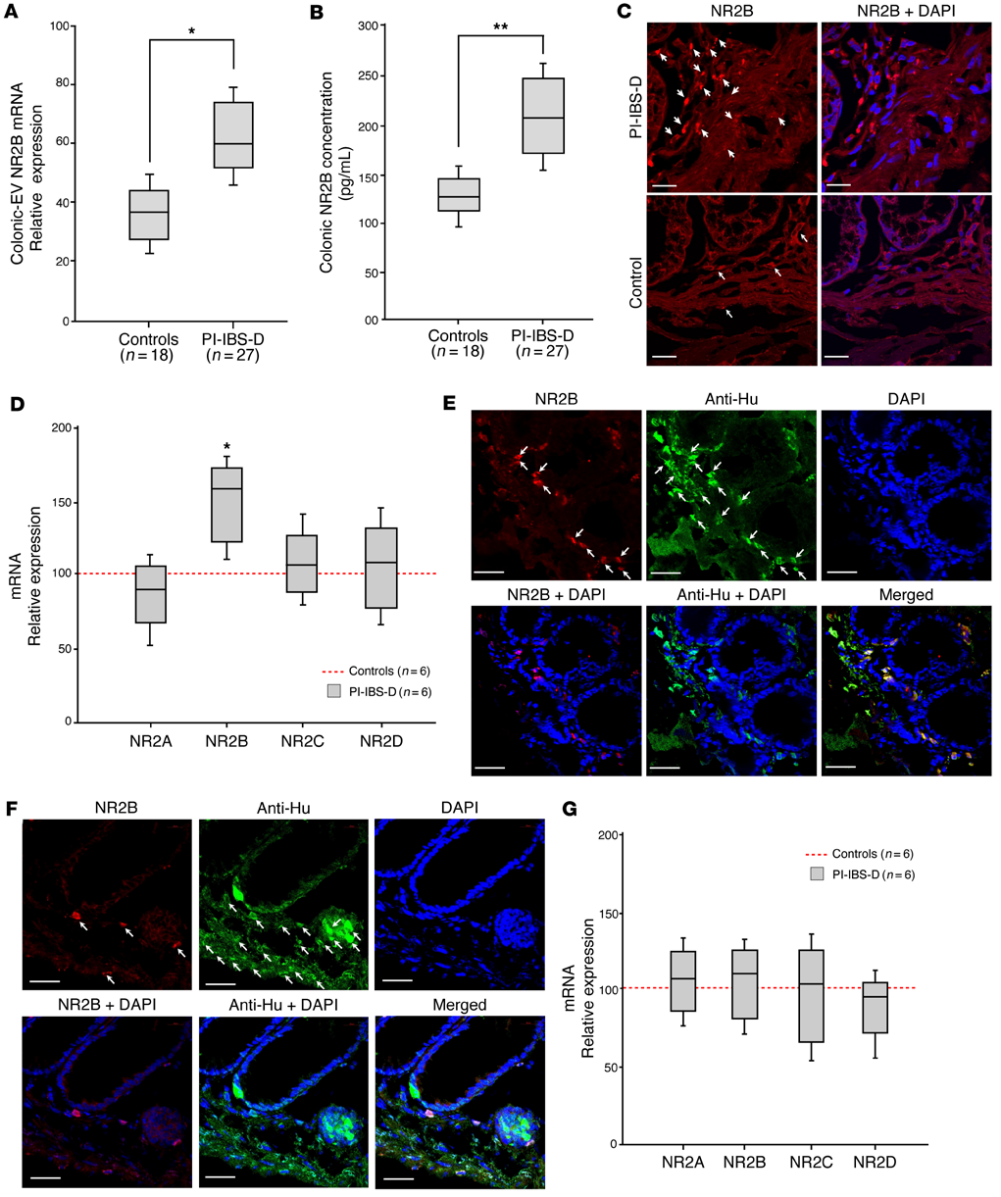
NMDA NR2B expression in colonic EVs, colon tissue, and cells in patients with PI-IBS-D. (**A**) Real-time PCR shows enhanced NMDA NR2B expression in colon EVs from patients with PI-IBS-D compared with control participants. (**B**) ELISA revealed enhanced colonic tissue NMDA NR2B expression in patients with PI-IBS-D versus control participants. (**C**) IHC images of NMDA NR2B expression (white arrows) in the colon tissue of patients with PI-IBS-D versus control participants. Scale bars: 50 μm. (**D**) Significantly increased mRNA expression of NMDA NR2B expression (but not NR2A, NR2C, or NR2D) in colonic neuronal cells in patients with PI-IBS-D versus control participants. (**E**) IHC showing NMDA NR2B colocalized with a neuronal marker (anti-Hu) (white arrows) in colon tissue from patients with PI-IBS-D. Scale bars: 40 μm. (**F**) IHC showing NMDA NR2B colocalized with a neuronal marker (anti-Hu) (white arrows) in colon tissue from control participants. Scale bars: 40 μm. (**G**) No differences in NMDA NR2A, NR2B, NR2C, and NR2D expression were observed in colonic epithelial cells of patients with PI-IBS-D versus control participants. *P = 0.05, **P = 0.01 by unpaired *t* test.

As NMDA NR2B was identified as a target of miR-23a/b, NMDA NR2B expression was evaluated in colonic EVs using real-time PCR. Colonic EV NMDA NR2B expression was higher in patients with PI-IBS-D than in the control participants (Figure 3A). Significantly increased levels of NMDA NR2B expression in patients with PI-IBS-D compared with control participants were also revealed by ELISAs and immunocytochemistry (IHC) of colon biopsy samples (Figure 3, B and C, respectively). Spearman’s correlation analysis revealed that NMDA NR2B expression in colonic EVs and colon tissues of patients with PI-IBS-D was positively correlated with the VAS scores for abdominal pain (*r* = 0.836, *P* = 0.001 and *r* = 0**.**748, *P* = 0.001, respectively).

Next, an analysis was performed to determine the colonic cells that might play a role in miR-23a/b and NR2B signaling. Laser capture microdissection was used to procure colonic neurons by using neuronal markers (anti-Hu) under direct microscopy. Only NR2B expression was upregulated (*P* = 0.05) in the colonic neurons of patients with PI-IBS-D (Figure 3D). Only minor changes were observed in the NR2A, NR2C, and NR2D expression levels. NR2B expression was colocalized with the anti-Hu neuron marker in colon tissues from patients with PI-IBS-D (Figure 3E) and control participants (Figure 3F). Laser capture microdissection was used to procure colonic epithelial cells using the CK20 epithelial marker. No significant differences in the expression of NMDA NR2 subtypes were observed in colonic epithelial cells between patients with PI-IBS-D and the control participants (Figure 3G). Other miR-23a/b targets, such as CXCR4 and CXCL12, were examined using laser capture microdissection to procure colonic neurons and epithelial cells. We found that CXCR4 and CXCL12 were upregulated not only in colon neuronal cells, but also in colon epithelial cells in patients with PI-IBS-D compared with control participants (Supplemental Figures 6 and 7). In this study, we focused on miR-23a/b and NR2B in colonic neuronal cells and dorsal root ganglia (DRGs) via colonic EV communication associated with abdominal pain. More details about the CXCR4/CXCL12/miR-23ab axis will be analyzed in future studies; this axis may be linked to the postinfectious cytokine-relevant pathway. We also tested NR2B expression in human colon biopsies by enteric pathogen group. Supplemental Figure 8 shows subgroups of patients with PI-IBS-D with a history of *Campylobacter* infection (*n* = 16), *Salmonella* infection (*n* = 6), and Shiga toxin–producing *E*. *coli* infection (*n* = 5). There was no significant difference in NR2B expression among groups.

### Evidence for in vitro endocytosis of colonic EVs and neuronal cells

#### Expression of miR-23a/b and NR2B in neuronal cells in an in vitro endocytic bioenvironment.

**Figure 4:**
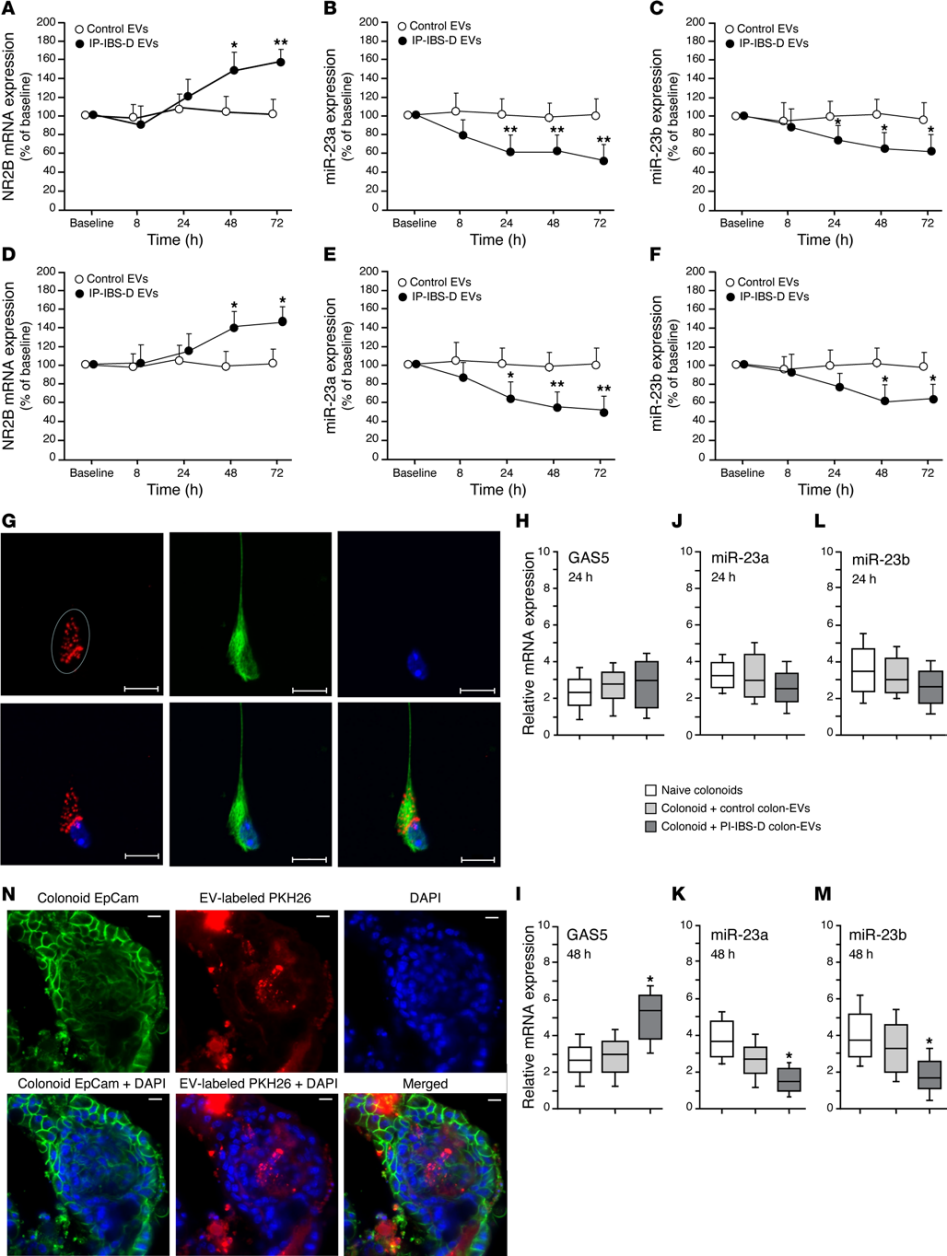
Evidence for in vitro endocytosis of colonic EVs and neuronal cells. (**A**) Coincubation of human neuronal cells (LUHMES, ATCC CRL-2927) with colonic EVs from patients with PI-IBS-D revealed increased NMDA NR2B expression starting at 24 hours and reaching significance at 48 hours. (**B** and **C**) Coincubation of human neuronal cells with colonic EVs from patients with PI-IBS-D lead to significantly diminished miR-23a (**B**) and miR-23b (**C**) after 24, 48, and 72 hours versus control participants. (**D**) Coincubation of mouse DRG cells with colonic EVs from patients with PI-IBS-D resulted in significantly increased NMDA NR2B expression at 48 and 72 hours. (**E** and **F**) Coincubation of mouse DRGs with colonic EVs from patients with PI-IBS-D lead to decreased miR-23a expression (**E**) at 24, 48, and 72 hours and decreased miR-23b expression (**F**) at 48 and 72 hours. (**G**) IHC showing endocytosis of human colonic EVs (red), neuronal cells (green), and DAPI (blue) after 8 hours of incubation. Scale bars: 10 μm. (**H**–**M**) Relative GAS5 (**H** and **I**), miR-23a (**J** and **K**), and miR-23b (**L** and **M**) expression in human colonoids incubated for 24 and 48 hours with colonic EVs from patients with PI-IBS-D or control participants. At 48 hours, GAS5 was significantly increased, and miR-23a and miR-23b were decreased. (**N**) Endocytosis of human colonic EVs (red) into the colonoids after 48 hours of incubation. Epithelial cell marker (EpCam, green) and EVs labeled with PKH26 (red). Scale bars: 10 μm. *P = 0.05, **P = 0.01 by unpaired *t* test.

Human neurons were coincubated with colonic EVs from patients with PI-IBS-D for 8, 24, 48, or 72 hours, after which miR-23a/b and NMDA NR2B expression were evaluated. NMDA NR2B expression increased starting at 24 hours, but this increase was not significant until 48 hours (Figure 4A). In contrast, decreased miR-23a (Figure 4B) and miR-23b (Figure 4C) expression was observed after 24, 48, and 72 hours of coincubation. Second, colonic EVs from patients with PI-IBS-D were coincubated with mouse DRGs for 8, 24, 48, or 72 hours, after which miR-23a/b and NMDA NR2B expression were evaluated. NMDA NR2B expression was upregulated after 48 hours and 72 hours of coincubation (Figure 4D). Additionally, miR-23a and miR-23b expression was significantly decreased in mouse DRGs coincubated with colonic EVs from patients with PI-IBS-D starting at 24 and 48 hours, respectively (Figure 4, E and F). Following the time course of RT-PCR experiments, endocytosis was demonstrated using confocal microscopy. After 8 hours of coincubation, endocytosis of human colonic EVs (red) into neuronal cells in cell culture was observed (Figure 4G).

#### Expression of miR-23a/b and GSA5 in human colonoids under in an in vitro endocytic bioenvironment.

Noncoding RNAs, including lncRNAs and miRNAs, have been shown to be key regulators of gene expression in the gut and may mediate enteric neural and intestinal epithelial damage following insults to the GI tract (23–26). To identify therapeutically targetable mechanisms in patients with PI-IBS-D and upstream regulators of the miR-23 family, we examined growth arrest–specific 5 (*GAS5*), a multismall nucleolar RNA host gene located on chromosome 1q25.1. GAS5 is a long noncoding RNA (lncRNA) that is a competing endogenous RNA that can obliterate or absorb miRNAs and adjust or restrain the biological functions of miRNAs, such as miR-23a/b (27–29). Human colonoids were coincubated with colonic EVs from patients with PI-IBS-D or control participants for 24 and 48 hours to evaluate GAS5 and miR-23a/b expression. The expression of GAS5 increased starting at 24 hours (Figure 4H) and reached significance at 48 hours (Figure 4I). In contrast, a slight decrease in miR-23a/b expression was observed starting at 24 hours (Figure 4, J and L) and a moderate reduction in expression was observed at 48 hours (Figure 4, K and M). After 48 hours of incubation, EV endocytosis into human colonoids was apparent (Figure 4N).

### In vivo induction of visceral hypersensitivity following i.p. injection of colonic EVs into mice

**Figure 5:**
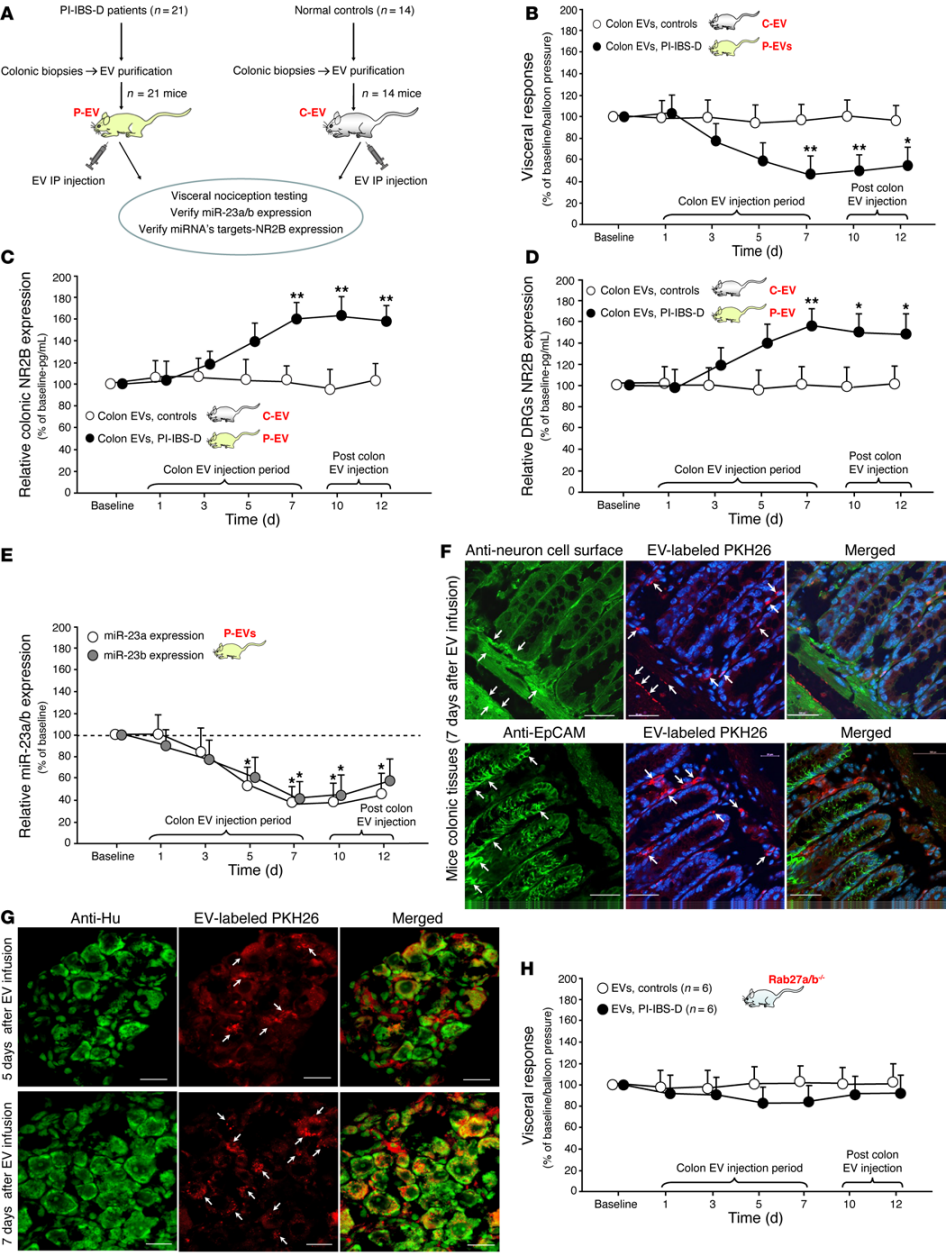
In vivo induction of visceral hypersensitivity following i.p. injection of colonic EVs into mice. (**A**) Schematic of the experimental design. (**B**) i.p. injection of PI-IBS-D colonic EVs (P-EVs) significantly increased VMR to CD compared with injection of control EVs (C-EVs). (**C**) Significantly increased NMDA NR2B (ELISA) expression was observed in the P-EV mouse colon (day 7–12) compared with the C-EV mouse colon. (**D**) Significantly increased NMDA NR2B (ELISA) expression was observed in P-EV mouse DRGs (day 7–12) versus those from C-EV mice. (**E**) MicroRNA-specific PCR assay indicated significantly decreased DRG miR-23a expression on days 5–12 after injection and significantly decreased miR-23b expression on days 7–12 after injection. (**F**) IHC in mouse colonic tissues showing human colonic EVs (labeled with PKH26) and colocalization with neuronal surface marker (A2B5-106) and epithelial surface marker (anti-EpCAM; ab71916). Scale bars: 50 μm. (**G**) IHC in mouse DRGs following injection of human colonic EVs: colonic EVs (labeled PKH26) and neuronal marker (anti-Hu). Scale bars: 20 μm. (**H**) No significant changes in visceral nociception were observed in Rab27a/b^–/–^ mice following i.p. injection of colonic EVs from patients with PI-IBS-D. *P = 0.05, **P = 0.01 by unpaired *t* test.

Given these results, the next series of experiments was conducted to determine whether EVs from patients with PI-IBS-D carry the factors necessary to trigger visceral hypersensitivity in vivo. EVs were purified from human colon tissues and human colonic EVs were injected i.p. into individual mice (Figure 5A). A total of 21 mice received colonic EVs from 21 individual patients with PI-IBS-D (P-EV mice), while 14 mice received colonic EVs from 14 control participants (C-EV mice). Visceral nociception testing was performed by measuring the visceromotor response (VMR) to the colorectal distension (CD). i.p. injection of human colonic EVs into mice resulted in a significant increase (~50%) in the VMR to CD in P-EV mice compared with that in C-EV mice, starting on day 5 and reaching statistical significance on day 7 (Figure 5B). This change was not observed in C-EV mice. This trend continued on days 10 and 12 after injection (Figure 5B). Upregulation of colonic NMDA NR2B expression relative to baseline occurred in the colons of P-EV mice starting on day 7 and continuing until day 12; this upregulation was not observed in C-EV mice (Figure 5C). DRG expression of NMDA NR2B followed a similar pattern, with increased expression of NMDA NR2B in mouse DRGs at 7, 10, and 12 days after colon EV injection in P-EV mice (Figure 5D) but not in C-EV mice. Moreover, DRG miR-23a expression decreased relative to baseline at 5, 7, 10, and 12 days in P-EV mice, whereas DRG miR-23b expression decreased at 7, 10, and 12 days (Figure 5E). To determine the colocalization of the injected EVs, colon tissues from P-EV mice were examined by microscopy 7 days after injection and labeled with a neuronal surface marker (anti-neuronal cell surface antigen antibody, clone A2B5-106, ref. 7), an epithelial surface marker (anti-epithelial cell adhesion molecule antibody, anti-EpCAM; ab71916), and an EV marker (PKH26) (Figure 5F). Similar staining analyses were performed for DRGs in P-EV mice at 5 and 7 days after injection (Figure 5G).

We divided the colon EVs from patients with IBS-D by enteric pathogen group before i.p. injection of colonic EVs into individual mice, such as EVs-miR-23a/b and NR2B expression, to determine whether there were individual differences between enteric pathogen groups. We found no significant differences in miR-23a/b and NR2B expression between the subgroups of patients with PI-IBS-D with a history of *Campylobacter* infection (*n* = 14), *Salmonella* infection (*n* = 3), or Shiga toxin–producing *E*. *coli* infection (*n* = 4) (Supplemental Figures 9 and 10).

To confirm the mechanistic role of EVs in PI-IBS-D progression, Rab27a/b^–/–^ mice deficient in EV secretion were used to verify EV functionality (Supplemental Figure 11). Following the injection of colonic EVs from patients with PI-IBS-D, Rab27a/b^–/–^ mice exhibited a slight, nonsignificant increase in visceral nociception, as determined by assessing the VMR to the CD (Figure 5H). Since Rab27a/b^–/–^ mice do not produce EVs, these findings indicate that EVs may function as important internal messengers in patients with PI-IBS-D. These data further suggest that colonic EVs from patients with PI-IBS-D evoke visceral nociception in WT mice via EV miR-23/NMDA NR2B communication between colonic and neuronal cells (i.e., DRGs). Rab27a/b–/– mice injected with EVs from PI-IBS-D mice did not develop visceral hypersensitivity, indicating that EVs are essential internal messengers.

### miR-23 oligonucleotides reverse visceral nociception via EV delivery

**Figure 6:**
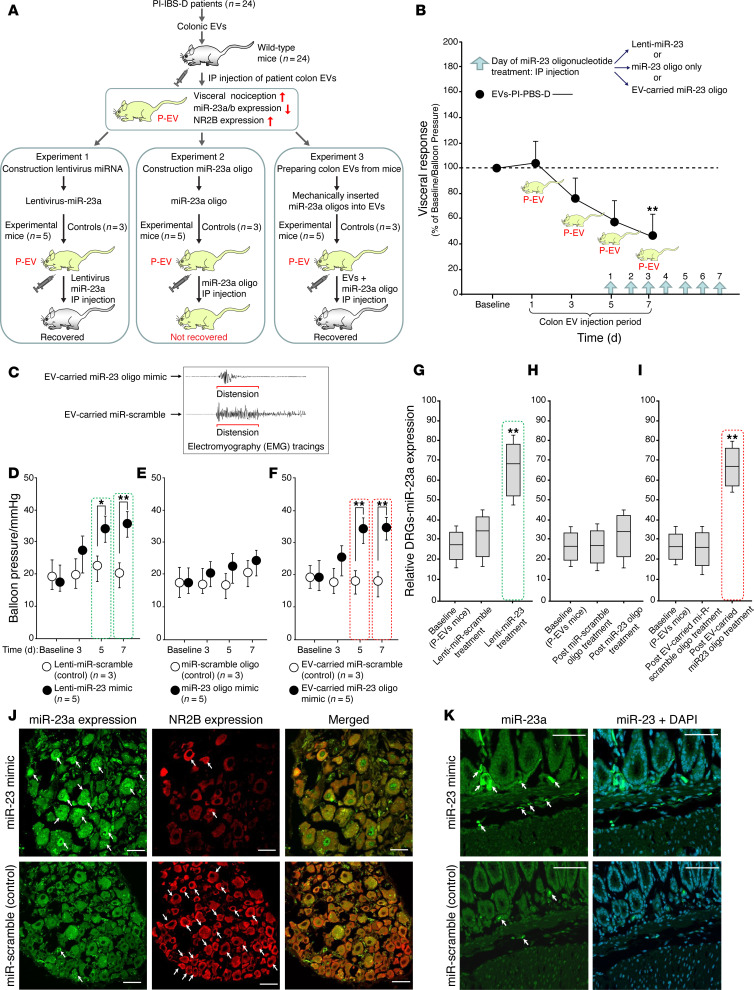
Therapeutic approach for miR-23 oligonucleotide–mediated reversal of visceral nociception via EV delivery. (**A**) Schematic diagram of overall experimental design. (**B**) Experimental design of miR-23 treatment in P-EV mice. Light blue arrows indicate oligo treatment days. (**C**–**F**) miR-23 oligo treatment in P-EV mice. (**C**) Increased EMG activity in P-EV mice after EV-carried miR-scramble (control) versus miR-23 oligo mimic treatment. (**D**) Significant decrease in the VMR to CD 5 days after lenti-miR-23a mimic treatment versus lenti-miR-scramble (control). (**E**) No significant change in VMR to CD after transfection with the miR-23a oligo mimic only. (**F**) P-EV mice that received miR-23 oligonucleotide mimic (without lentivirus as vehicle) with an EV carrier showed a large reduction in visceral hypersensitivity at days 5 and 7 versus EV-carrier-scrambled miR (control). (**G**) Significant enhancement of DRG miR-23a in P-EV mice after treatment with lenti-miR-23 but not with (**H**) miR-23a oligo. (**I**) Treatment of P-EV mice with EV-carried miR-23 oligo leads to a significant increase in miR-23a. (**J**) Double labeling of the mouse DRGs using FISH and IHC. Top: Increased miR-23a expression and decreased NMDA NR2B expression following EV-carried oligo-miR-23a mimic. Merged image shows colocalization of miR-23a and NMDA NR2B expression in the mouse DRG. Bottom: EV-carried miR-scramble (control) decreased miR-23a expression and increased NMDA NR2B expression. Merged image shows colocalization of miR-23a and NMDA NR2B expression in the mouse DRG. Scale bars: 50 μm. (**K**) miR-23a (FISH) expression in mouse colon tissues after EV-carrying oligo-miR-23a mimic (top) treatment versus miR-scramble (control) (bottom). Scale bars: 40 μm. *P = 0.05, **P = 0.01, by unpaired *t* test.

Three separate experiments (Figure 6A) were performed to determine whether miR-23 oligonucleotides delivered by EVs could reverse visceral nociception. First, 24 mice received colonic EVs from 24 individual patients with PI-IBS-D (P-EV mice) and were divided into 3 groups (*n* = 8 per group). P-EV mice received an i.p. injection of lenti-miR-23 (*n* = 5) or lenti-miR-scramble (*n* = 3) as a control (see Experiment 1 in Figure 6), or received miR-23 oligonucleotides only (*n* = 5) or an miR-scramble oligonucleotide only as a control (*n* = 3) (see Experiment 2 in Figure 6). In the third group, miR-23 oligonucleotides were mechanically inserted into purified human EVs (EV-miR23 oligonucleotides), which were then i.p. injected into P-EV mice (*n* = 5) or into an miR-scrambled oligonucleotide as a control (*n* = 3) (see Experiment 3 in Figure 6). The VMR to CD ratio was measured. The detailed experimental design for all 3 experiments is shown in Figure 6A.

On day 5 of the 7-day EV injection period, i.p. injections of a lenti-miR-23-oligonucleotide (mimic), miR-23 oligonucleotide only, or EV-miR-23-oligonucleotides (EV carrying miR-23 oligo) were administered along with the corresponding control treatments to the P-EV mice, as described above (Figure 6A). On day 5 (7 days after EV injection), lenti-miR-23-oligo (mimic) was injected (i.p.) into P-EV mice or human EV-miR-23 oligo (purified human EV, then miR-23 oligos were pressed into human EVs) (Figure 6B). Significant decreases in the VMR to CD were observed after 5 days of treatment with the lenti-miR-23 mimic compared with the lenti-miR-scramble (control) (*P* = 0.05; Figure 6A, Experiment 1, and Figure 6D). No significant reduction in the VMR to CD was observed after i.p. injection with miR-23 oligonucleotides only (Figure 6A, Experiment 2, and Figure 6, C and E) in the absence of an EV carrier. More importantly, in P-EV mice, EVs carried the miR-23 oligonucleotide (without lentivirus as a vehicle) and showed a large reduction in the VMR to CD after 5 days (*P* = 0.01) of injection with an miR-23 mimic with an EV carrier compared with the corresponding control (Figure 6A, Experiment 3, and Figure 6F).

A significant enhancement in DRG miR-23a expression was observed in P-EV mice after i.p. injection with lenti-miR-23 (Figure 6A, Experiment 1, and Figure 6G). No significant changes in expression were observed 7 days after i.p. injection with the miR-23 oligonucleotide only (Figure 6A, Experiment 2, and Figure 6H) without an EV carrier. There was enhanced expression of DRG miR-23a in P-EV mice following treatment with EV-carried miR-23 (Figure 6A, Experiment 3, and Figure 6I). EV-carried miR-23 oligos result in highly efficacious intracellular communication with neuronal cells, which provides significant translational opportunities for using EVs as therapeutic delivery vehicles. Next, double labeling of mouse DRGs 7 days after i.p. injection with the EV-carried miR-23 oligonucleotide was performed using FISH and immunocytochemistry. A labeled miR-23a–specific probe was used to visualize miR-23a expression and to determine if it colocalized with NR2B target gene expression. Increased miR-23a expression and decreased NMDA NR2B expression were observed in mice after 7 days of treatment with EV-carried oligo-miR-23a mimics in mouse DRGs (Figure 6J). Mice that received EV-carried miR-scrambled (control) treatment showed enhanced NMDA NR2B expression (Figure 6J, bottom panels). Using FISH, we also identified miR-23a expression in the colon tissues of mice that received 7 days of treatment with the EV-carrying oligo-miR-23a mimic (Figure 6K, top) but not in those of mice that received miR-scramble (control) (Figure 6K, bottom).

### lncRNA GAS5 contributes to visceral nociception through interactions with miR-23a/b

**Figure 7:**
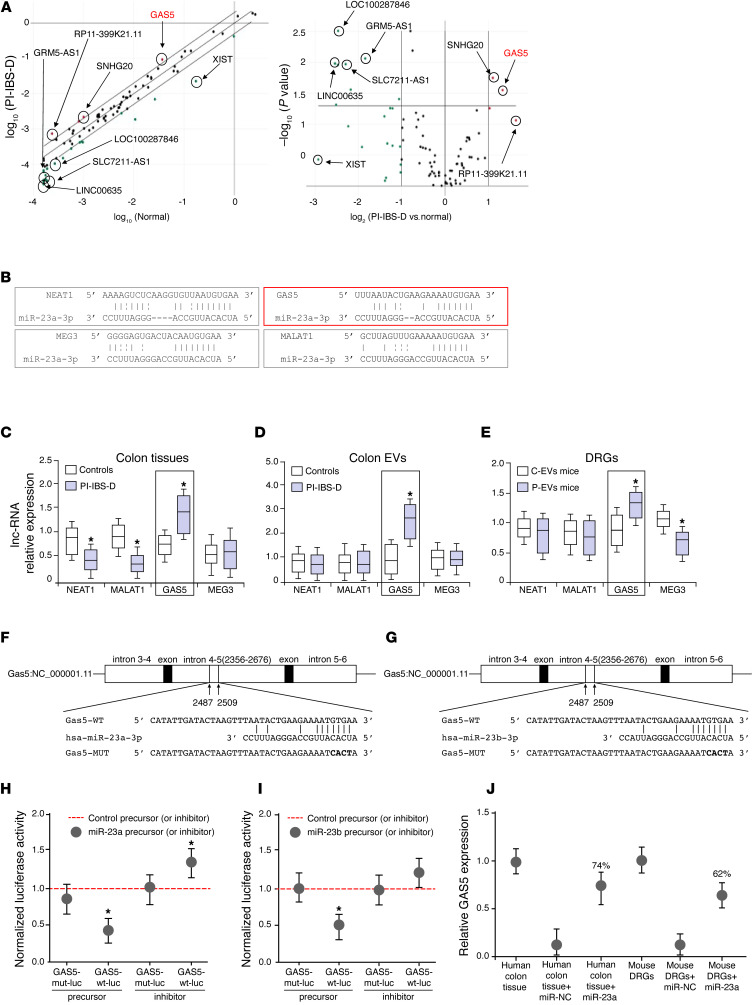
lncRNA GAS5 contributes to visceral nociception through interactions with miR-23a/b. (**A**) GAS5 and SNHG20 levels in PI-IBS-D colonic EVs are significantly increased (Human Inflammatory Response and Autoimmunity RT² lncRNA PCR Array). (**B**–**E**) Analysis using RegRNA 2.0 Gene prediction software: (**B**) lncRNAs NEAT1, MALAT1, GAS5, and MEG3 (but not SNHG20) had sequences complementary to miR-23a. RT-PCR showed significant GAS5 upregulation in both (**C**) colon tissues and (**D**) EVs of patients with PI-IBS-D versus control participants and that (**E**) GAS5 was also significantly upregulated in the DRGs of the P-EV mice versus C-EV mice. (**F**–**I**) Confirmation of the functional interaction between lncRNA GAS5 and miR-23a/b using a dual-luciferase assay. The relative luciferase activity of psiCHECK-2-GAS5-wt was reduced following cotransfection with oligo-miR-23a/b mimic, whereas that of oligo-miR-23a-NC and psiCHECK-2-GAS5-mut were unchanged. (**J**) Evaluation of the binding rate for miR-23a with GAS5 with an RNA pull-down assay showed that miR-23a pulled down 74% and 62% of GAS5 in human colon tissues and mouse DRGs, respectively.

Next, a series of experiments were performed to determine the upstream factors (s) that led to the downregulation of miR-23 expression in patients with PI-IBS-D. Using the Human Inflammatory Response and Autoimmunity RT² lncRNA PCR Array (Qiagen), the expression levels of two lncRNAs, GAS5 and SNHG20, were significantly increased in colonic EVs from patients with PI-IBS-D (Figure 7A and Supplemental Figure 12). Additionally, the 2**.**0 gene prediction software RegRNA 2.0, was used to predict lncRNAs with miR-23 as the potential target gene. Four lncRNAs (NEAT1, MALAT1, GAS5, and MEG3) were predicted to have sequences complementary to miR-23 (Figure 7B). The expression of these lncRNAs in colon tissues and colonic EVs from patients with PI-IBS-D was assessed using real-time PCR (Figure 7, C–E). lncRNA GAS5 expression was significantly upregulated in the colon tissue and colonic EVs of patients with PI-IBS-D compared with that in control participants (Figure 7, C and D). GAS5 was selected for the current study because it competes with miR-23a/b for binding sites. DRGs harvested from P-EV and C-EV mice revealed that GAS5 was the only lncRNA significantly upregulated in DRGs from P-EV mice compared with those from C-EV mice (*P* = 0.05; Figure 7E).

To investigate whether lncRNA GAS5 binds to miR-23, a dual-luciferase assay was performed. The relative luciferase activity of psiCHECK-2-GAS5-wt was reduced after cotransfection with the oligo-miR-23a/b mimic, whereas those of oligo-miR-23a-NC and psiCHECK-2-GAS5-mut remained unchanged (Figure 7, F–I). RNA pull-down assay was performed to assess the binding of miR-23a to GAS5 relative to the total amount of GAS5. The results indicated that miR-23a pulled down 74% and 62% of GAS5 in human colon tissue and mouse DRGs, respectively (Figure 7J).

**Figure 8:**
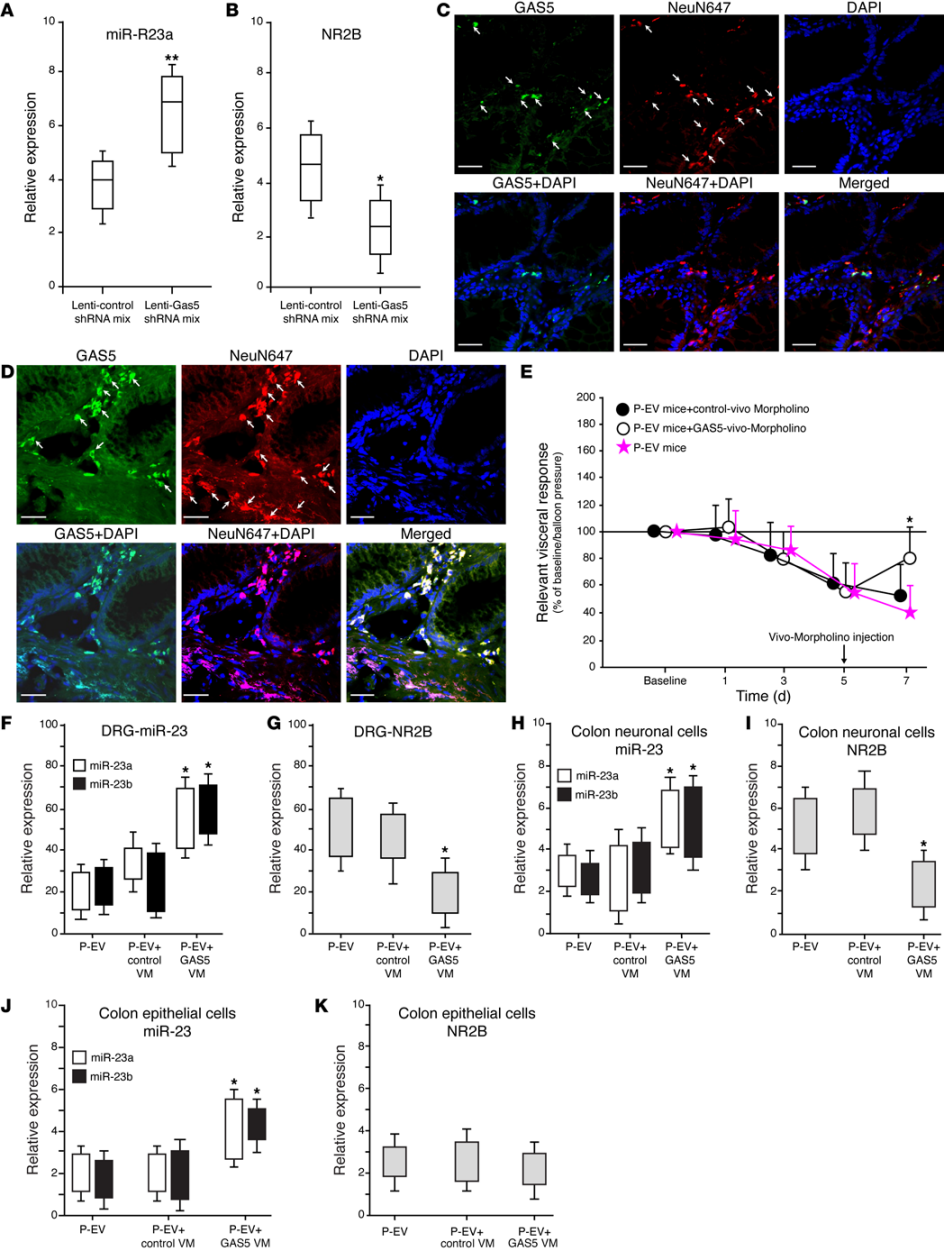
Silencing or knockdown of GAS5 regulated miR-23 and NR2B expression. (**A**) Silencing of lncRNA GAS5 increases the expression level of miR-23a, indicating that lncRNA GAS5 directly binds to miR-23a in DRG neurons. (**B**) NMDA NR2B expression was significantly downregulated after silencing of lncRNA GAS5. (**C**) FISH with IHC in PI-IBS-D colon tissue showing GAS5 colocalized with a neuronal marker (NeuN647). (**D**) FISH with IHC in PI-IBS-D colon tissue showing GAS5 colocalized with NMDA NR2B. (**E**–**K**) Vivo-Morpholino (VM) knockdown of GAS5. (**E**) P-EV mice administered GAS5-Vivo-Morpholino on day 5 showed significantly decreased VMR to CD compared with that of controls. (**F**) Increased miR-23a/b expression in mouse DRGs 2 days following GAS5-Vivo-Morpholino. (**G**) Decreased NMDA NR2B expression in P-EV mouse DRGs 2 days following GAS5-Vivo-Morpholino. (**H** and **I**) Single-cell PCR in colonic neuronal cells revealed significantly increased miR-23a/b expression (**H**) but decreased NMDA NR2B expression (**I**) in colonic neuronal cells after i.p. injection with GAS5-Vivo-Morpholino. (**J** and **K**) Single-cell PCR in colonic epithelial cells showed significantly increased miR-23a/b expression (**J**) but not NR2B (**K**) after GAS5-vivo-Morpholino. *P = 0.05, **P = 0.01 by unpaired *t* test.

lncRNAs and miRNAs can regulate each other and participate in the occurrence and development of a variety of human diseases, including IBS and IBD, by forming a complex molecular regulatory network (29–31). A series of experiments have demonstrated that GAS5 targets miR-23 and negatively regulates its expression and that GAS5 is the only lncRNA significantly upregulated in DRGs from P-EV mice compared with C-EV mice. Next, lncRNA GAS5 genes in human neuronal cells (LUHMES) were silenced using lentiviral transduction to investigate the interactions among GAS5, miR-23, and NMDA NR2B. Silencing lncRNA GAS5 increased the expression of miR-23a (Figure 8A), indicating that lncRNA GAS5 directly binds to miR-23a in the DRG neurons. In contrast, NMDA NR2B expression was significantly downregulated after silencing of lncRNA GAS5 (Figure 8B). Moreover, GAS5 colocalized with a neuronal marker (Figure 8C) and NMDA NR2B (Figure 8D) in the colon tissues of patients with PI-IBS-D. These data suggest that, in patients with PI-IBS-D, higher levels of GAS5 may alter NMDA NR2B function by inhibiting miR-23 expression in colonic neuronal cells, subsequently increasing visceral nociception.

Vivo-Morpholinos (Gene Tools) were designed against the highly conserved regions of GAS5 to block the putative splice sites in vivo. To examine the GAS5/miR-23/NMDA NR2B axis in further detail, we knocked down GAS5 function in vivo to determine whether GAS5 modulates NMDA NR2B expression via miR-23a/b expression and whether GAS5-Vivo-Morpholino can be used to treat P-EV mice (Figure 8, E–K). A pair of morpholinos was generated by targeting GAS5 in the mice. P-EV mice received i.p. injections of EVs for 7 days and we administered GAS5-Vivo-Morpholino via i.p. injection on day 5. Two days after GAS5-Vivo-Morpholino treatment, a significant decrease in VMR to CD (*P* = 0.05) was observed compared with the controls (Figure 8E). Decreases in NMDA NR2B expression in P-EV mice were observed in mouse DRGs 2 days after i.p. injection with GAS5-Vivo-Morpholino (Figure 8G); these decreases were accompanied by increased miR-23a/b expression (Figure 8F). Single-cell PCR in colonic neuronal cells revealed significantly increased miR-23a/b expression in colonic neuronal cells after i.p. injection with GAS5-Vivo-Morpholino, accompanied by decreased NMDA NR2B expression (Figure 8, H and I). miR-23a/b expression significantly increased in colonic epithelial cells (Figure 8J), whereas NMDA NR2B expression remained unchanged (Figure 8K). Taken together, these results indicate the mechanistic role of GAS5, which when knocked down by Vivo-Morpholino, decreases NMDA NR2B expression and increases miR-23a/b, leading to a reduction in visceral nociception in P-EV mice.

## Discussion

The findings of our current study show that colonic EVs mediate cell-cell communication in patients with PI-IBS-D and act as biological messengers that transfer noncoding RNAs to key intestinal signaling pathways that drive persistent gastrointestinal dysfunction following enteric infections. To our knowledge, our findings are the first to show the following. (a) Colonic EVs from patients with PI-IBS-D exhibited decreased miR-23a/b and increased NMDA NR2B levels (32–36), and miR-23a/b was inversely correlated with VAS abdominal pain scores. (b) Translational datasets (in vitro, in vivo, and human ex vivo) demonstrated endocytoplasmic communication between human colonic EVs and enteric neurons; this communication resulted in an increased VMR to CD in mice via upregulation of NMDA NR2B expression (37, 38) but not in mice that are EV deficient (Rab27a/b^–/–^). (c) We identified mechanistic links between upregulation of NR2B expression and downregulation of miR-23a/b expression, leading to experimentally induced visceral nociception. (d) In vivo manipulation (upregulation) of miR-23 expression through administration of miR-23a oligonucleotides (packaged in EVs) decreased visceral nociception and diminished NMDA NR2B signaling. (e) lncRNA GAS5 competed for binding sites with miR-23 and decreased miR-23 expression, resulting in increased NR2B expression via the GAS5/miR-23/NMDA NR2B axis. (f) GAS5 played a key mechanistic role in P-EV mice, which when knocked down by Vivo-Morpholino, decreased NMDA NR2B expression and increased miR-23a/b, which reduced visceral nociception.

We believe the results of this study are new for several reasons. At a basic scientific level, they constitute a potentially new understanding of the roles of lncRNAs, miRNAs, and NMDA receptors in postinflammatory enteric dysfunction (Supplemental Figure 13). They also contribute to our understanding of the regulation of colonic visceral nociception in the pathophysiology of PI-IBS-D. Most importantly, this study has important clinical significance. These translational findings may lead to the development of new targeted therapies for patients with PI-IBS-D using EVs as internal delivery vehicles. Silencing GAS5 using Vivo-Morpholino upregulated miR-23a/b expression, which decreased the NR2B signaling pathway and led to decreased visceral nociception (39–42).

Although interindividual differences in miRNA expression were observed in EVs isolated from patients with IBS and control participants, they were not statistically significant, suggesting modest variability. In the P-EV animal model, in which patient-derived EVs were administered to mice, this variability was accounted for despite some individual differences, which likely did not significantly affect the overall findings, enabling a clearer assessment of EV-derived miRNAs in PI-IBS-D pathology because we focused only on miR-23a/b and NR2B in the current study. These findings advance our current understanding of how colonic EVs facilitate and drive chronic gastrointestinal dysfunction in patients with PI-IBS-D following enteric infections. Colonic injuries to epithelial cells caused by enteric infections may induce cellular communication with enteric neurons as well as with the peripheral nervous system (along the DRGs) via colonic EVs, which potentiate and propagate chronic gastrointestinal symptoms, resulting in increased visceral nociception via the GAS5/miR-23/NMDA NR2B axis pathway (Supplemental Figure 13). Thus, EVs act as internal biological vehicles to deliver treatments, as we have shown with the mechanical insertion of the miR-23 oligo, which reverses visceral nociception in mice.

In conclusion, our findings suggest that blocking lncRNA GAS5 or augmenting miR-23 expression in colonic EVs from patients with PI-IBS-D can inhibit or even reverse gastrointestinal dysfunction and nociception by downregulating NMDA NR2B expression. Moreover, these findings suggest a provocative theory that colonic EVs travel to distant extraintestinal sites in humans where they modulate downstream targets. More interestingly, colonic EVs may be one mechanism underlying the extraintestinal symptoms commonly observed in patients with IBS. The findings of the current study were supported by the experimental approaches used in this translational study: in vivo, in vitro, and ex vivo. These translational findings may lead to the development of novel treatments using EVs as internal delivery vehicles to carry miRNA oligonucleotides and internal messages, thereby treating patients with intractable gastrointestinal dysfunction following enteric infections.

## Methods

### Sex as a biological variable.

Both male and female patients and mice were used for all of the studies and considered in the analysis.

Patients (ages 18–72 years) who met the Rome IV criteria for IBS-D, had symptoms for >5 years, had a documented case of enteric infection, and were diagnosed with PI-IBS-D were included in the study.

All participants underwent (a) a comprehensive history and physical examination, (b) a breath test for bacterial overgrowth, and (c) a tissue transglutaminase antibody titer. Patients with a history of IBD, microscopic colitis, lactose intolerance, bacterial overgrowth, chronic intestinal pseudo-obstruction, celiac sprue, pancreatitis, cirrhosis, or food allergies were excluded from the study.

Qualified participants also completed an abdominal pain questionnaire, with a standardized, 0–100 VAS scale in which patients rated their level of abdominal pain on a continuous line marked from 0 (no pain) to 100 (the most severe pain imaginable). All participants underwent colonoscopy, and random biopsies were obtained using Jumbo biopsy forceps (Radial Jaw4, Boston Scientific) from the colon for hematoxylin and eosin staining to exclude inflammation and for molecular and ncRNA studies.

### Mice.

Details regarding the experimental methods are available in the Supplemental Methods and include (a) isolation of human colonic EVs, (b) miRNA microarray and PCR array profiling, (c) lncRNA PCR array profiling, (d) cell culture, (e) flow cytometry, (f) in vivo studies, and (g) statistical methods. The treatment days for the mouse experiments were carefully selected based on the typical progression of IBS-like symptoms and timing of physiological responses to the administered EV-derived miRNAs. Previous studies have shown that a treatment duration of 12 days is optimal to observe significant changes in gut motility and immune responses, which are critical indicators of IBS pathology. This time frame also aligns with established protocols for inducing and monitoring IBS symptoms in murine models, ensuring that the effects of the treatment are accurately captured.

### Statistics.

All statistical analyses were performed using GeneSpring GX software (version 7.3, Agilent Technologies), Prism (version 6, GraphPad Inc.), and ASA software (version 9.1.3). One-way ANOVA was performed, followed by Tukey’s comparison or Benjamini and Hochberg correction for false-positive reduction. Two-tailed Student’s *t* tests were also used. Data are shown as the mean ± SD. Human tissue samples were paired for comparisons based on age- and sex-matching. Pearson’s correlation coefficients were calculated to explore the association between miR-23a/b, GAS5, and NR2B.

### Study approval.

The human studies were approved by the Institutional Review Boards of Tulane University and the University of Tennessee. All participants signed an informed consent form before the start of the study. Animal studies were approved by the Institutional Animal Care and Use Committees of Tulane University and the University of Tennessee and were performed according to NIH guidelines.

### Data availability.

Values for data points in graphs are reported in the Supporting Data Values file. Requests for further information should be directed to the corresponding author.

## Author contributions

QZ provided conceptualization, analyzed and interpreted data, drafted and edited the manuscript, and obtained funding. LY analyzed and interpreted data and conducted experiments. ZTV and BBZ analyzed the data and conducted experiments. JZF edited the manuscript. ATT reviewed the manuscript. GNV provided conceptualization, analyzed and interpreted data, drafted and edited the manuscript, and obtained funding.

## Supplementary Material

Supplemental data

Supporting data values

## Figures and Tables

**Table 1:**
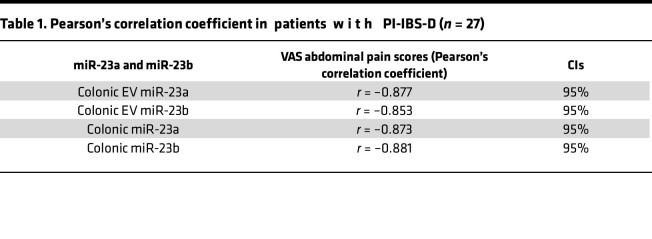
Pearson’s correlation coefficient in patients with PI-IBS-D (*n* = 27)

## Version 1 03/10/2025

Electronic publication
