# Supplementary material for: Human colonic EVs induce murine enteric neuroplasticity via the lncRNA GAS5/miR-23/NMDA NR2B axis
Source: JCI Insight. 2025 Mar 10;10(5):e178631. doi: 10.1172/jci.insight.178631 (PMC11949048; doi:10.1172/jci.insight.178631)
Supplement: Supplemental data [file jciinsight-10-178631-s323.pdf]

SUPPLEMENTARY METHODS

Human colon tissue EV extraction protocol:

1) Tissues with cold PBS, homogenized with handheld homogenizer at 60% speed (1st time) for 1 minute, centrifuged at 15,000g at 4°C for 10 min. Supernatant collected.
2) Add cold PBS to pellet, homogenize at 100% speed (2nd time) for 1 minute, centrifuge at 15,000g at 4°C for 10 min, collect supernatant, keep on ice. An appropriate amount of cold PBS was added to the pellet, homogenized at 100% speed (3rd time) for 1 minute, centrifuge at 15,000g at 4°C for 10 min, collect supernatant.3ml of cold PBS added to supernatant, transfer to ultra-centrifuge tube, centrifuge at 200,000g for 70min at 4°C.
3) Pellet dispersed in ultra-centrifuge tube with 100μl cold PBS, and transferred the suspension to 1.5mL Eppendorf tube on ice.
4) Rinse the ultra-centrifuge tube with 100μL cold PBS, combine with previous 100μL suspension (200μL final volume).
5) Centrifuge at low speed briefly (2,000g at 4°C for 1 minute) to pellet the protein.
6) Recover the 200μL of supernatant which contains EVs ready for injection or other analysis.

Quantitative Real-Time PCR Assay:

Total RNA was extracted with mirVana™ miRNA Isolation Kit (Invitrogen cat#: AM1560). For mRNA assays, reverse transcription was done using 1μg of total RNA and RT^2 First Strand Kit (Qiagen Cat#: 330401). mRNA PCR was done using RT^2 SYBR Green qPCR Mastermix (Qiagen Cat#: 330500) and gene specific primer kit, RT^2 qPCR Primer Assay (Qiagen Cat#: 330001). The Reaction mixture was incubated for 10 min at 95°C and the process was repeated for 40 cycles (denaturing for 15s at 95°C and annealing and extending for 1 min at 60°C) using Eppendorf Mastercyler ep realplex 2 according to the manufacturer's protocol. For miRNA assay, reverse transcription was done using 100ng of total RNA and TaqMan MicroRNA Reverse Transcription Kits (Applied Biosystems, Cat#: 4366596). miRNA RT-PCR was done with miRNA gene specific TaqMan MicroRNA Assays kit (Applied Biosystems cat#: 4440886). The Reaction mixture was incubated for 2 min at 95°C and the process was repeated for 40 cycles (denaturing for 15s at 95°C and annealing for 15s at 55°C and extending for 20s at 68°C) using Eppendorf Mastercyler ep realplex 2 according to the manufacturer's protocol. GAPDH and U6 was used as an endogenous reference for mRNA and miRNA respectively. The comparative C_T method ($\Delta\Delta C_T$)was used for quantification of gene expression. All samples were test in triplicate, and average values used for quantification.

Cell Culture Studies:

Human LUHMES cells were cultured according to ATCC's protocol. Briefly, culture flasks were pre-coated with 50μg/mL poly-L-ornithine and then with 1μg/mL human fibronectin. Cells were seeded at $2x10^6$ per T-75 flask or $4x10^5$ per well of 6-well plate. Cells were cultured with ATCC-formulated DMEM:F12 Medium (Catalog No. 30-2006) supplemented with 1% N2 supplement (Gibco-Invitrogen Cat No 17502-048) and 40 ng/ml b-FGF (basic recombinant human Fibroblast Growth Factor; Gibco-Invitrogen Cat No 13256-029). Cells were incubated at 37°C with 5% CO_2.

DRG neural cell culture: Papain (Worthington, cat. no. 3126) solution, Collagenase type II (CLS2) (Worthington, cat. no. 4176) / Dispase type II (MB, cat. no. 165859) solution was prepared

according to the Nature Protocol (Nature Protocol 2 (1):152-60, February 2007). The mouse DRG dissection and DRG cell preparation were performed according to the Nature Protocol. Briefly, 6-8week old mice were sacrificed according to protocol and the spinal column was excised and cut into halves. Spinal cord was removed and DRGs were dissected with fine forceps. DRGs were kept in 3 mL ice cold Hank's balanced salt solution without Ca^{++}/Mg^{++} (HBSS, Thermo Fisher, 14170-112) in 60 mm x 15 mm petri dishes until all DRGs were collected. The axon bundles found on the outside of the DRG were cut away using fine spring scissors under a dissection microscope (VWR SZB250, Cat #630-1577). DRGs and HBSS were transferred to collection tubes. We removed 1.5 ml of HBSS followed by the addition of papain solution to tissue/HBSS in collection tubes and incubated for 10 min in a 37 °C water bath. Samples were spun to pellet ganglia and the papain solution was removed. Collagenase/dispase solution was added to tissue in collection tubes and incubated for 10 min in a 37 °C water bath. Tissue was spun to remove collagenase/dispase solution. We then added 2 ml of prewarmed culture medium (F12 with 10% FCS, penicillin/streptomycin) to tissues. Tissue was spun briefly to form pellets and the medium was removed. Then 0.5 ml of culture medium was added to tissues in tubes. Samples were triturated using pipette until the solution became cloudy. Cells were plated on laminin/poly-D-lysine-coated coverslips
(Corning® BioCoat™ Cat# 354087). Then 0.7 ml of culture medium was added to triturated DRGs and mixed. Next, 100 µl cell suspension was carefully pipetted on to each laminin/poly-D-lysine-coated coverslip (in wells). Plates were placed in a 37°C, 5% CO_2 incubator.

Transfections and Transduction:

HumanGas5 shRNA vector and control shRNA vector were purchased from Origene (Rockville, MD). Human Gas5 shRNA lentivirus and control shRNA lentivirus were packaged with Lenti-vpak packaging kit (OrigeneCat# TR30037). Pre-miRNA precursors lenti-virus (Lenti-miR-23a), negative control lentivirus (lenti-control-miR) and transduction reagent Transdux (SBI cat# LV850A-1) were purchased from System Biosciences (SBI, Palo Alto, CA). Transfections were carried out using Lipofectamine® 2000 Transfection Reagent (Invitrogen Cat#: 11668030) according to the manufacturer's guidelines. Lentivirus transduction was carried out with 4ug/mL polybrene(SCBT Cat#:SC-134220).

Mouse DRG cells: GAS5 shRNAlentivirus (Lenti-GAS5) and control shRNA lentivirus (Lenti-control) (Origene, Rockville, MD). Lipofectamine 2000 (Invitrogen cat# 11668-027). Lenti-miR-23a, lenti-miR-control (SBI, Palo Alto, CA). miR-23a and miRNA control (Ambion), mirVana miRNA isolation kit (Ambion cat# AM1560). GAS5 shRNA lentivirus transduction of LUHMES cells. LUHMES cells were plated in 6-well plates and cultured according to the ATCC's protocol. Transduction was done when cells reached 70% confluency. Briefly, 24 hours prior to transfection, in a 6-well tissue culture plate seed 4 x 10^5 cells in 3 ml of standard growth medium per well. Grow cells to a 70% confluency. For each well transfection, 4ug/mL polybrene and lenti-virus was added to each well at MOI of 20 and swirled to mix. Change medium without Polybrene after 24 hours. Continue culture for another 48 hours (72 hours in total post transduction), the viral genome was integrated into the host cell genome.

Fluorescence In situ RNA hybridization (FISH):

A locked nucleic acid (LNA) probe with complementarity to Human GAS5 was labeled with 5′and 3'-digoxigenin and synthesized by Qiagen (Germantown, MD, USA). A scrambled LNA probe was used as a negative control. The slides were prehybridized for 30 min at 52°C and then 10 pmol of the probe in 60-100 µl of hybridization mixture was added to each slide and incubated for 1 h at 52°C. Slides were incubated with 3% (vol/vol) H_2O_2 for 10 min at room temperature (RT) to block endogenous peroxidases before applying HRP conjugated antibodies. After washing,

slides were incubated in blocking buffer for 30 minutes at RT. Then 100-150 ul antibody was added and incubated for 30 minutes at RT. The slides were washed again at RT on a shaker. TSA™ Plus Fluorescein System (PerkinElmer, Waltham, MA, USA) was used for direct fluorescence detection according to the manufacturer's protocol. Finally, 25 µl of 'Prolong Gold containing DAPI' was added to each section and the whole slide was covered with glass coverslips. The slides were imaged using an epifluorescence microscope equipped with a charge-coupled device camera and image analysis.

Laser capture microdissection (LCM) of human colonic tissues:

Tissue was treated in 10% formalin overnight and embedded in paraffin blocks. Slide cutting was then done with RNase AWAY® solution. Specimens were cut into 7 µm sections and floated using a 42°C water bath. Sections were allowed to flatten. Each section was mounted at room-temperature onto PEN Membrane Glass Slides (ThermoFishercat#: LCM0522, KIT0312). The section was then immediately deparaffinized, stained and, dehydrated according to the protocol or were mounted in stored slides at -70°C for future use. Laser capture microdissection (LCM) with an ArcturusXT™ microdissection system was then performed according to the manufacturer's protocol. CapSure® Macro LCM Caps (ThermoFishercat# LCM0211) and an infrared (IR) laser was used to cut tissue to prevent UV damage to DNA and RNA in the samples. The dissected samples were immediately processed or stored them at -70°C. For RNA extraction and isolation, we followed the manufacturer's protocol (ThermoFishercat#: Paradise PLUS Extraction RA7001, RA7007). For RNA amplification, we followed the manufacturer's protocol (ThermoFishercat# : Paradise® PLUS cDNA kit - RA7018, In Vitro Transcription (IVT) 2-round – RA7009).

Isolation of mouse colon epithelial cells by laser capture microdissection

Frozen colon tissue sections were cut with 6 µm thickness from OCT frozen blocks and placed on PEN-membrane RNase-free slides (Leica Microsystems, Buffalo Grove, IL). After colon tissues were fixed in 100% ice-cold acetone for 10 min, colon epithelial cells were stained by anti-CK20 primary antibody (1:100, Thermo Fisher Scientific, Waltham, MA) for 1 hr at 4°C and Cy2-conjugated secondary antibody (1:100, Jackson ImmunoResearch Laboratories, West Grove, PA) for 1 hr at 4°C. CK20 epithelial cells were dissected and collected by LCM using Leica LMD7000 (Leica Microsystems). Total RNA from collected macrophages were isolated using PicoPure RNA isolation kit (Thermo Fisher Scientific).

Isolation of mouse colon neurons by laser capture microdissection

Frozen colon tissue sections were cut with 6 µm thickness from OCT frozen blocks and placed on PEN-membrane RNase-free slides (Leica Microsystems, Buffalo Grove, IL). After colon tissues were fixed in 100% ice-cold acetone for 10 min, colon neurons were stained by anti-HuC/D primary antibody (1:100, abcam) for 1 hr at 4°C and Cy2-conjugated secondary antibody (1:100, Jackson ImmunoResearch Laboratories, West Grove, PA) for 1 hr at 4°C. HuC/D neurons were dissected and collected by LCM using Leica LMD7000 (Leica Microsystems). Total RNA from collected macrophages were isolated using PicoPure RNA isolation kit (Thermo Fisher Scientific).

Confocal Fluorescence Microscopy:

Cells were fixed in 4% paraformaldehyde, permeabilized in 0.1% Triton X-100, and probed withMouse-anti-CD68 and rabbit-anti-NR1 antibody (1:500; ABCam) at the same time. The primary antibodies were detected using donkey-anti-mouse-Alexa 488 and donkey-anti-rabbit Alexa 594 conjugated antibodies (1:1000; Invitrogen). Prolong Gold containing DAPI was added to each section and the whole slides were covered with glass coverslips. The slides were imaged using Zeiss 710 confocal microscope. Images were analyzed with Zen Blue software.

Antibodies combinations (1:500):
1) CD68(mouse, abcam ab955) + NR2B(rabbit, abcam ab65783)
2) Pan-neuron (mouse, milliporeMAB2300) + NR2B(rabbit)
3)NR2B (mouse, abcam ab93610) + Anti-HuC/D(rabbit, abcam ab184267)

Luciferase reporter assay
(1) Human GAS5 and miR-23a/b luciferase assay
Intact putative GAS5 recognition sequences from the 5′- region of miR-23a or with random mutations were cloned downstream of the *Renilla* luciferase reporter gene of psiCHECK-2 vector. 293T cells were plated in 24-well plate 24 hours prior transfection. Cells were cotransfected with 1 μg of WT-GAS5 or MUT-GAS5 constructs and 30pmol of miR-23a/b, miR-23a/b inhibitor, or control-miRNA, control-miRNA inhibitor using transfection reagent. Luciferase assays was performed 48h after transfection using the Dual-Luciferase Reporter Assay system (Promega, Madison, WI).

(2) NR2B and miR-23a/b luciferase assay
Intact putative NR2B recognition sequences from the 5′- region of miR-23a/b or with random mutations were cloned downstream of the *Renilla* luciferase reporter gene of psiCHECK-2 vector. 293T cells were plated in 24-well plate 24 hours prior transfection. Cells were cotransfected with 1μg of WT-NR2B or MUT-NR2B constructs and 30pmol of miR-23a/b, miR-23a/b inhibitor, or control-miRNA, control-miRNA inhibitor using transfection reagent. Luciferase assays was performed 48 h after transfection using the Dual-Luciferase Reporter Assay system (Promega, Madison, WI).

(3) CXCL12 and miR-23a/b luciferase assay
Intact putative CXCL12 recognition sequences from the 5′- region of miR-23a or with random mutations were cloned downstream of the *Renilla* luciferase reporter gene of psiCHECK-2 vector. 293T cells were plated in 24-well plate 24 hours prior transfection. Cells were cotransfected with 1μg of WT- CXCL12 or MUT- CXCL12 constructs and 30pmol of miR-23a/b, miR-23a/b inhibitor, or control-miRNA, control-miRNA inhibitor using transfection reagent. Luciferase assays was performed 48 h after transfection using the Dual-Luciferase Reporter Assay system (Promega, Madison, WI).

(4) CXCR4 and miR-23a/b luciferase assay
Intact putative CXCR4 recognition sequences from the 5′- region of miR-23a or with random mutations were cloned downstream of the *Renilla* luciferase reporter gene of psiCHECK-2 vector. 293T cells were plated in 24-well plate 24 hours prior transfection. Cells were cotransfected with 1μg of WT- CXCR4 or MUT- CXCR4 constructs and 30pmol of miR-23a/b, miR-23a/b inhibitor, or control-miRNA, control-miRNA inhibitor using transfection reagent. Luciferase assays was performed 48 h after transfection using the Dual-Luciferase Reporter Assay system (Promega, Madison, WI).

Human GAS5 knockdown assay:
(1) Human GAS5 knock down by shRNA
GAS5 shRNA lentivirus transduction of LUHMES cells. LUHMES cells were plated in 6-well plates and cultured according to the ATCC's protocol. Transduction was done when cells reached 70% confluency. Briefly, 24 hours prior to transfection, in a 6-well tissue culture plate seed 4 x 10^5 cells in 3 ml of standard growth medium per well. Grow cells to a 70% confluency. For each

well transfection, 4ug/mL polybrene and lenti-virus (Lenti-GAS5 and Lenti-control) was added to each well at MOI of 20 and swirled to mix. Change medium without Polybrene after 24 hours. Continue culture for another 48 hours (72 hours in total post transduction), the viral genome was integrated into the host cell genome. The medium was removed and each well was washed with PBS (at this point the plate was frozen at -80C for future use), proceeded with RNA extraction using mirVana kits according to the manufacturer's protocol for gene expression assay.

(2) Human GAS5 knockdown by miR-23a

LUHMES cells were plated in 6-well plates and cultured according to the ATCC's protocol. Transduction was done when cells reached 70% confluency. Briefly, 24 hours prior to transfection, in a 6-well tissue culture plate seed 4 x 10^5 cells in 3 ml of standard growth medium per well. Grow cells to a 70% confluency. For each well transfection, 4ug/mL polybrene and lenti-virus (Lenti-miR-23a and Lenti-miR-control) was added to each well at MOI of 20 and swirled to mix. Change medium without Polybrene after 24 hours. Continue culture for another 48 hours (72 hours in total post transduction), the viral genome was integrated into the host cell genome. The medium was removed and each well was washed with PBS (at this point the plate was frozen at -80C for future use), proceeded with RNA extraction using mirVana kits according to the manufacturer's protocol for gene expression assay.

Visceromotor Response Testing:

A Flat Bottom Holder (Kent Scientific Corp, Torrington, CT) plastic restrainer was used to hold the unsedated mice during colonic distension. Visceral hypersensitivity testing was done by using electrodes (Teflon-coated stainless-steel wire, 5- to 10-mm tip separation; Cooner Wire Sales, Chatsworth, CA) that are sewn into the external oblique abdominal muscles of the mice to perform the electromyographic (EMG) recordings to measure the Visceromotor Response (VMR) following colorectal distension. Surgeries to implant the EMG electrodes took place at least 2 weeks before colorectal distension to allow time for healing and recovery.

EMG activity was quantified 10 s before colonic distension; 20 s during colonic distension; and 10 s after colonic distension. Colonic distension testing was done following a 12 hour fast. A balloon (1 cm long, 0.5 cm max diameter) made of polyethylene was secured to tubing attached to an automated distension device (G&J Electronic Inc., Toronto, Canada) was used to perform colonic distension. The balloon was lubricated and placed into the mouse's distal colon so that the tip of the balloon is 1 cm from the anus and allowed to acclimatize for 10 minutes before testing started. Using an automated distension device, the mice received phasic distension (0-80 mmHg in 5 mmHg ascending increments of 10 seconds each) of the colon and the Visceromotor Response (VMR) for each distension was recorded. The colonic distensions were repeated 3 times and the mean, Visceromotor Response for each pressure was recorded. The minimal pressure that elicits an EMG response was defined as the visceral sensitivity threshold.

Methods for EV (dose/concentration) injection (IP) into mouse:

Each colonic biopsy (jumbo forceps) retrieves ~15 mg of tissue for a total of ~225 mg of colon tissue for each set of biopsies taken per patient. The ~225 mg of colon tissue yields 1,080 ug (1.08mg) of EVs per patient. This provided 150 ug of EVs (~62.5 billion particles) in each dose that were injected into each mouse daily for a total of 7 days.

Mouse DRG EV was labeled with PKH26 Red Fluorescent Cell Linker Kit (Sigma Ca#018K1718) according to manufacturer's protocol. Labeled EV was suspended with 200uL of sterile PBS and injected into BL/6 mise through IP. Wait for 4 days and 7 days, Mice were euthanized and colon and DRG were collected. Tissued were treated with 10% formalin and 0.5M sucrose, mount in

OCT followed by frozen cutting. Colon slides were counterstained with Epicam(epithelial cells primary antibody, Abcam cat# ab71916), Alexa fluor 488(secondary antibody, green dye) and DAPI. DRG slides were counterstained with pan-neuron (neuron cells primary antibody, Millipore cat# MAB2300), Alexa fluor 488(secondary antibody, green dye) and DAPI. Slides were observed under confocol microscope.

Methods for Colonic-EV via endocytosis into neuronal cells:

Mouse DRG cells was prepared as mentioned earlier. DRG cells were plated on laminin/poly-D-lysine-coated coverslips in 12-well plate and cultured for 4-7 days. 5ug of mouse DRG EV which was labeled with PKH26 Red Fluorescent Cell Linker Kit was added to medium and incubated for 3-7 days. DRG cells on glass coverslips were rinsed 3x with PBS and counterstained with pan-neuron (neuron cells primary antibody, Millipore cat# MAB2300), Alexa fluor 488(secondary antibody, green dye) and DAPI. Slides were observed under confocal microscope.

Human colonoid culture protocol:

Human colonoids were routinely passaged following the protocol with slight modifications (Miyoshi et al. Nat Protoc). Fresh human colon tissues/biopsies were kept in cold saline or PBS before processing on the same day. Forceps were used to move the segment through the clean buffer to rinse thoroughly. PBS removed and tissue was rinsed with 15mL cold PBS again. Tissue was cut into 2mmx2mm fragments, transferred to 50mL tube and rinsed with cold PBS by pipetting up and down 3 times. Let tissue fragments sink to bottom and repeat the rinse step for 12 times. Tissue fragments were collected and 20mL of gentle cell dissociation reagent (STEMCELL, Catalog # 100-0485) was added for digestion. Tissue was digested by rotating at 20rpm at room temperature for 20 minutes. Resuspend Remove the digestion solution and transfer tissue fragments to fresh tube. 10 mL of cold PBS + 0.1% BSA was added and pipetted up and down for 3 times. Suspension was passed through 70 micron cell strainer and transferred to to a fresh 15 mL conical tube. Repeat the step for 4-5 times. Centrifuge at 200 x g for 3 minutes at 2 - 8°C. Gently pour off and discard the supernatants. Pelleted crypts will remain in the tubes. Resuspend each crypt pellet in 10 mL of cold (2 -8°C) DMEM/F-12 with 15 mM HEPES. Add 1 mL of each suspension to individual wells of a 6-well plate and assess the quality of the intestinal crypts. Centrifuge at 200 x g for 5 minutes at 2-8°C. Remove and discard the supernatant. Add 300 μL of complete IntestiCult Organoid Growth Medium (STEMCELL cat# 06010) at room temperature to each pellet. Add 300 μL of undiluted Matrigel to each tube and pipette up and down 10 times to resuspend the pellet. Avoid introducing bubbles. Carefully pipette 50 μL of the crypt suspension into each well of the pre-warmed 24-well plate. Incubate at 37°C for 20 minutes until the Matrigel is solidified. Add 750 μL of complete IntestiCult Organoid Growth Medium with 10μM ROCK inhibitor (Millipore, cat# SCM075) at room temperature to each well by pipetting the medium gently down the wall of the well.

Methods for loading of miR-23 oligos into to Colonic EVs by electroporation:

Colonic EVs were prepared as described earlier. After pelleting, the EVs were resuspended using pre-cold electrolytic buffer provided in Neon™ Transfection System 10 μL Kit (ThermoFisher Scientific). EVs were analyzed by nanoparticle tracking analysis (NTA), using the NanoSight NS300 system (Malvern, Cambridge, UK) according to the manufacturer's protocol. EVs and miRNA were mixed using the electroporation buffer at ratios of 3×10^9 EVs and 20 pmol of miRNA-23 oligo or control-miRNA oligo (Qiagen). The EV-miRNA mixtures were electroporated with 750V voltage and using a pulse width of 20 ms for 10 pulses, according to the manufacturer's protocol. Samples were then incubated for 30 min at 37 °C, followed by ultracentrifugation at 100,000 g for 2 hours at 4 °C to remove unbounded miRNAs, then resuspended in cold PBS. Electroporation of EVs in the absence of miRNAs was used as controls.

Supplementary References

Brumovsky PR, La JH, McCarthy CJ, et al. Dorsal root ganglion neurons innervating pelvic organs in the mouse express tyrosine hydroxylase. *Neuroscience* 2012;223:77-91.

Malin SA, Davis BM, Molliver DC. Production of dissociated sensory neuron cultures and considerations for their use in studying neuronal function and plasticity. Nat Protoc 2007;2:152-60.

Miyoshi H, Stappenbeck TS. In vitro expansion and genetic modification of gastrointestinal stem cells in spheroid culture. Nat Protoc 2013;8:2471-82.

Staab JF, Lemme-Dumit JM, Latanich R, et al. Co-Culture System of Human Enteroids/Colonoids with Innate Immune Cells. CurrProtoc Immunol. 2020 Dec;131(1):e113. doi: 10.1002/cpim.113. PMID: 33166041

Smith HS. In vitro properties of epithelial cell lines established from human carcinomas and nonmalignant tissue. J Natl Cancer Inst 1979;62:225-230.

Staab JF, Lemme-Dumit JM, Latanich R, et al. Co-culture system of human enteroids/colonids with innate immune cells. CurrProtoc Immunol 2020;131:e131

Supplementary Figure 1A

miR-23a/b Expression in Human Colon Biopsies

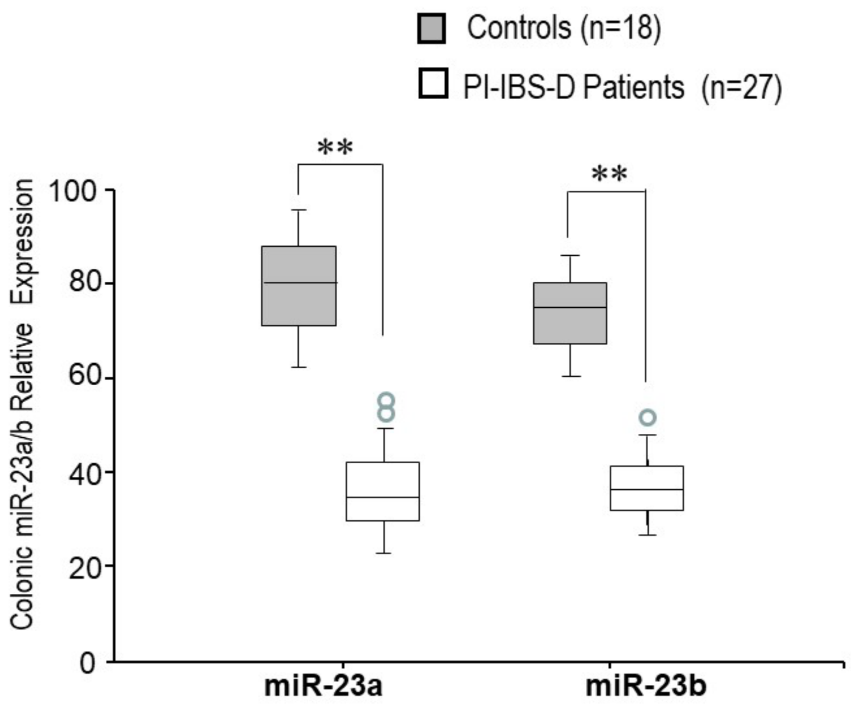

Supplementary Figure 1A. miR-23a/b-specific real-time PCR analysis of colon tissues from patients with PI-IBS-D. There was a similar miR-23a/b expression pattern in colon-EVs, which found that miR-23a/b was significantly downregulated in colon tissue from the same PI-IBS-D patients. (** $p<0.01$, vs. controls).

Supplementary Figure 1B

miR-155 Expression in Human Colon Biopsies & Colon-EVs

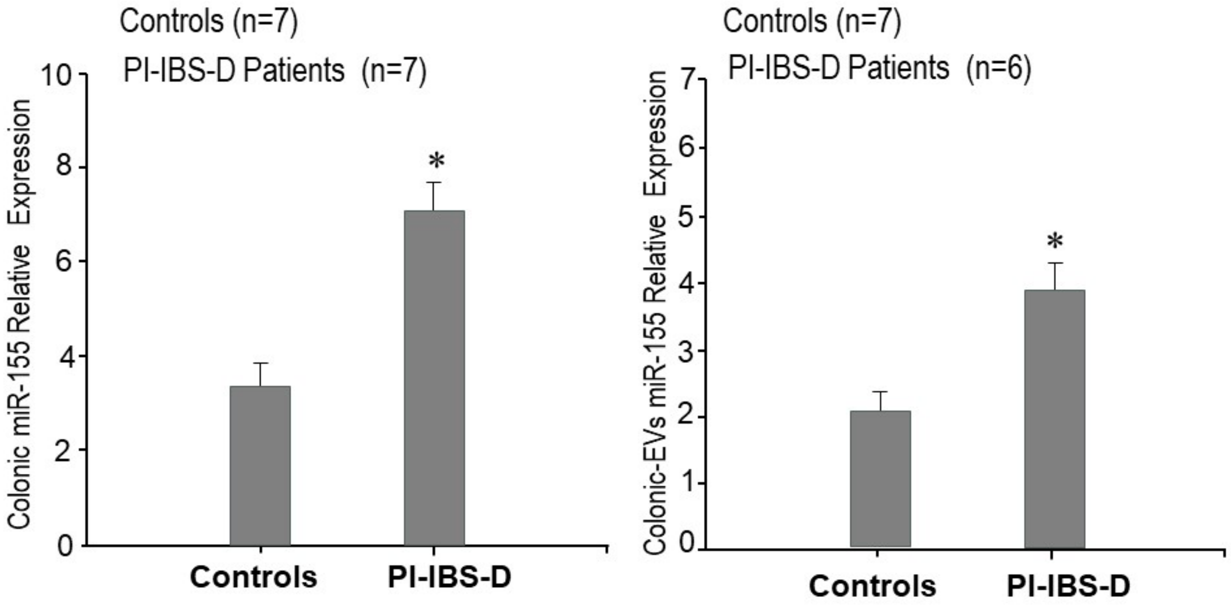

Supplementary Figure 1B. Real-time PCR analysis of miR-155 in colon tissues from patients with PI-IBS-D revealed significantly enhanced miR-155 expression not only in colon biopsy tissues of patients with PI-IBS-D but also in colon-EVs. (*p<0.05, vs. controls).

Supplementary Figure 2.

miR-23a/b Expression in PI-IBS-D patients' Colon Biopsies by Enteric Pathogen

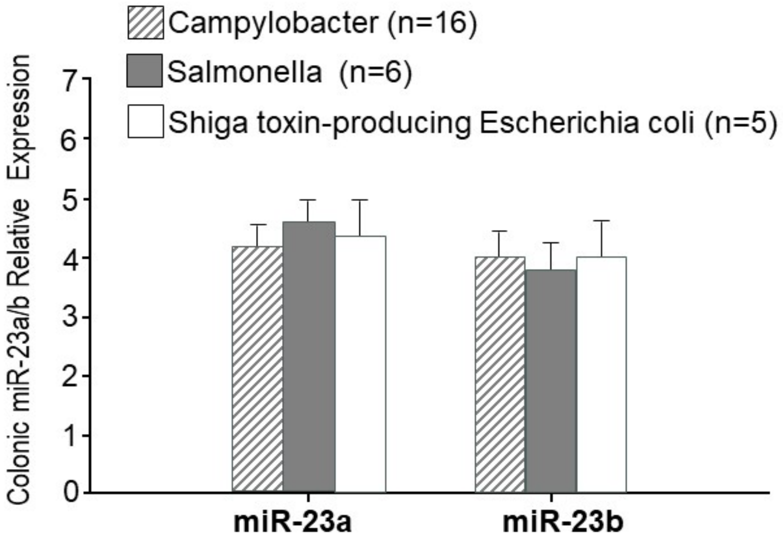

Supplementary Figure 2. miR-23a expression in colon biopsies of patients with PI-IBS-D infected by enteric pathogens. Sixteen PI-IBS-D patients had a Campylobacter infection history, 6 PI-IBS-D patients had a Salmonella infection history, and 5 PI-IBS-D patients had a Shiga toxin-producing Escherichia infection history. There was no significant difference in miR-23a/b expression between groups.

Supplementary Figure 3

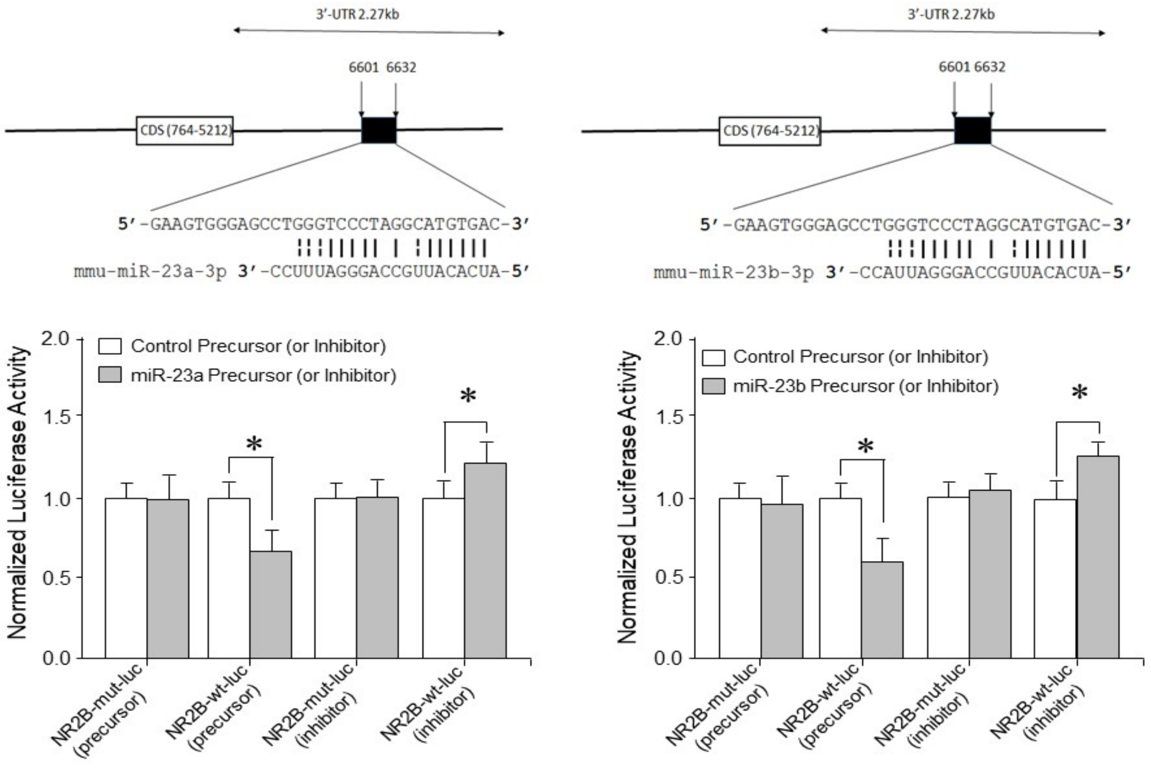

Supplementary Figure 3. Verification of the relationship between miR-23a/b and NR2B in mouse YAMC cells. Upper panel: The miRbase database indicates the presence of a highly conserved binding site for miR-23a/b in the 3'-UTR regions of the NMDA NR2B mRNA, a well-characterized Glutamate Receptor NMDA subtype. Further investigation of miR-23a/b revealed that Grin2B (NR2B) carries a putative miR-23a/b-binding site in mice. Lower panel: A dual-luciferase reporter assay with a vector containing the putative (mutant) 3'-UTR target site downstream of a luciferase reporter gene was designed. A highly conserved binding site for miR-23a/b in the 3'-UTR region of the NMDA NR2B mRNA was also seen in mice YAMC cells (*p< 0.05)

Supplementary Figure 4

```
Mouse CXCR4(188-208): 5'-UGUUUGUUUGUUUCAUGUGAA-3'
                                     ||||||
      mmu-miR-23a-3p: 3'-CCUUUAGGGACCGUUACACUA-5'
```

```
Mouse CXCR4(188-208): 5'-UGUUUGUUUGUUUCAUGUGAA-3'
                                     ||||||
      mmu-miR-23b-3p: 3'-CCAUUAGGGACCGUUACACUA-5'
```

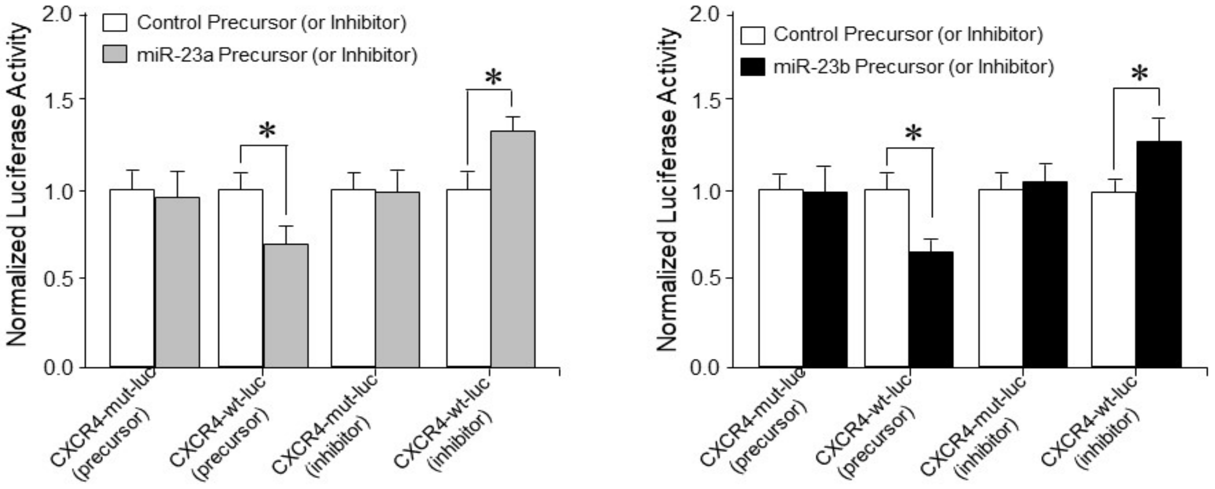

Supplementary Figure 4. Verification of the target relationship between miR-23a/b and CXCR4 in HEK293T cells. Upper panel: The miRbase database indicates the presence of a highly conserved binding site for miR-23a/b in the 3'-UTR regions of CXCR4 mRNA. Lower panel: A dual-luciferase reporter assay with the vector containing the putative (mutant) 3'-UTR target site downstream of a luciferase reporter gene was designed. A highly conserved binding site for miR-23a/b in the 3'-UTR region of CXCR4 mRNA was also observed in mouse HEK293T cells (*p< 0.05). The relative luciferase reporter activity of HEK293T cells co-transfected with miR-23a/b precursors and a psiCHECK-2-CXCR4-WT (wild-type) vector was significantly lower than that of HEK293T cells transfected with a psiCHECK-2-CXCR4-MUT (mutant) vector. In contrast, HEK293T cells transfected with psiCHECK-2-CXCR4-WT and miR-23a/b inhibitors displayed increased luciferase activity compared to those transfected with psiCHECK-2-CXCR4-MUT. Cell cultures were performed to test the mechanistic relationship between CXCR4 and miR-23a/b.

Supplementary Figure 5

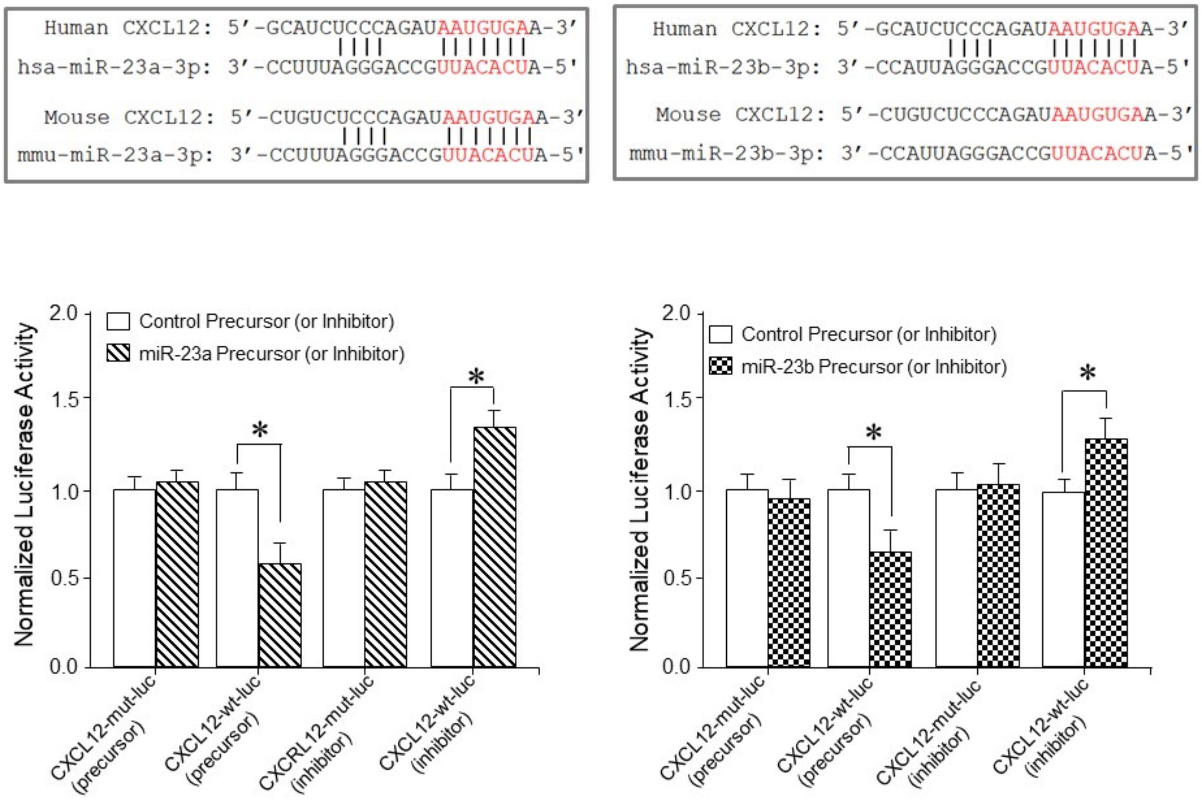

Supplementary Figure 5. Verification of the relationship between miR-23a/b and CXCL12 in HEK293T cells. Upper panel: The miRbase database indicates the presence of a highly conserved binding site for miR-23a/b in the 3'-UTR regions of CXCL12 mRNA. Lower panel: A dual-luciferase reporter assay with the vector containing the putative (mutant) 3'-UTR target site downstream of a luciferase reporter gene was designed. A highly conserved binding site for miR-23a/b in the 3'-UTR regions of CXCL12 mRNA was also observed in mouse HEK293T cells (*$p < 0.05$). The relative luciferase reporter activity of HEK293T cells co-transfected with miR-23a/b precursors and a psiCHECK-2-CXCL12-WT (wild-type) vector was significantly lower than that of HEK293T cells transfected with a psiCHECK-2-CXCL12-MUT (mutant) vector. In contrast, HEK293T cells transfected with psiCHECK-2-CXCL12-WT and miR-23a/b inhibitors displayed increased luciferase activity compared to those transfected with psiCHECK-2-CXCL12-MUT. Cell cultures were performed to test the mechanistic relationship between CXCL12 and miR-23a/b.

Supplementary Figure 6

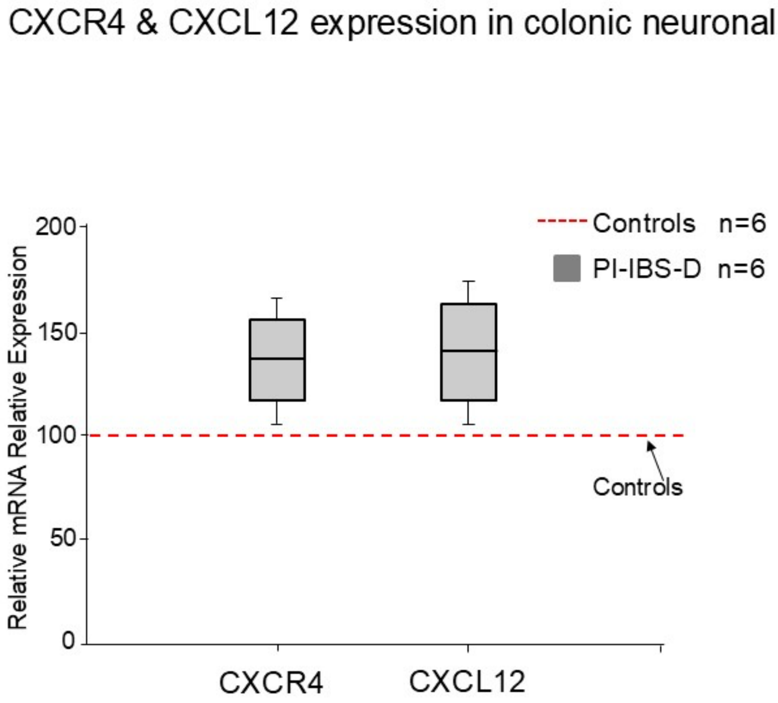

Supplementary Figure 6. CXCR4 and CXCL12 expression in colonic neuronal cells from colon biopsies of PI-IBS-D patients. There is no difference between CXCR4 and CXCL12 in colon-neuronal-cells from 6 PI-IBS-D. There was an increase in both CXCR4 and CXCL12 expression compared to that in the controls.

Supplementary Figure 7

NR2B CXCR4 & CXCR12 expression in colonic Epithelial cells

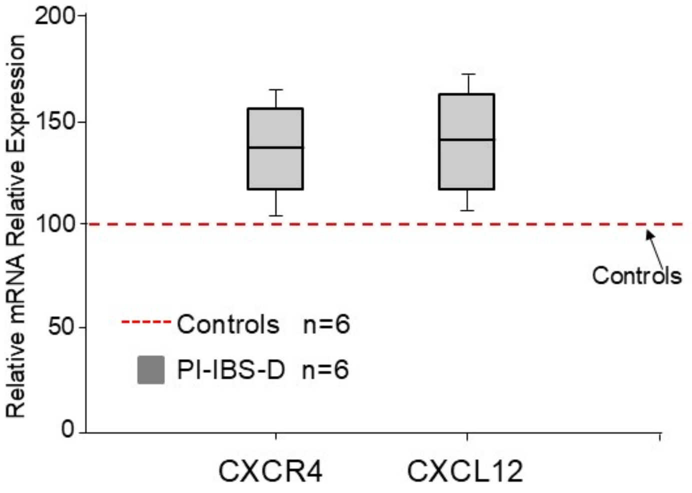

Supplementary Figure 7. CXCR4 and CXCL12 expression in colonic epithelial cells from colon biopsies of PI-IBS-D patients. There was no difference between CXCR4 and CXCL12 in colon-neuronal-cells from 6 PI-IBS-D. There was an increase in both CXCR4 and CXCL12 expression compared to that in the controls.

Supplementary Figure 8

NR2B Expression in PI-IBS-D Colon Biopsies by Enteric Pathogen

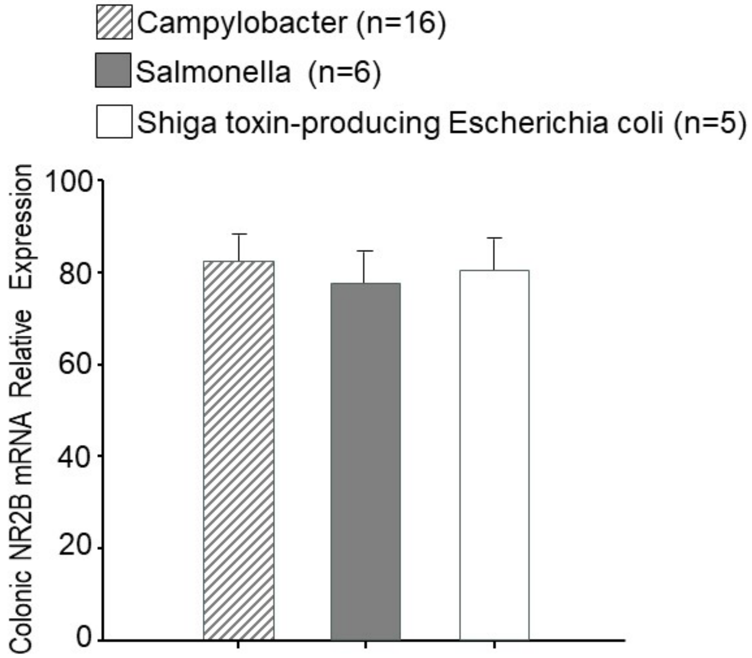

Supplementary Figure 8. NR2B expression in colon biopsies of PI-IBS-D patients infected by enteric pathogens. There were 16 PI-IBS-D patients with Campylobacter infection history, 6 PI-IBS-D patients with Salmonella infection history, and 5 PI-IBS-D patients with Shiga toxin-producing Escherichia infection history. There was no significant difference in miR-23a/b expression between the groups.

Supplementary Figure 9.

miR-23a/b Expression in PI-IBS-D patients' Colon-EVs by Enteric Pathogen

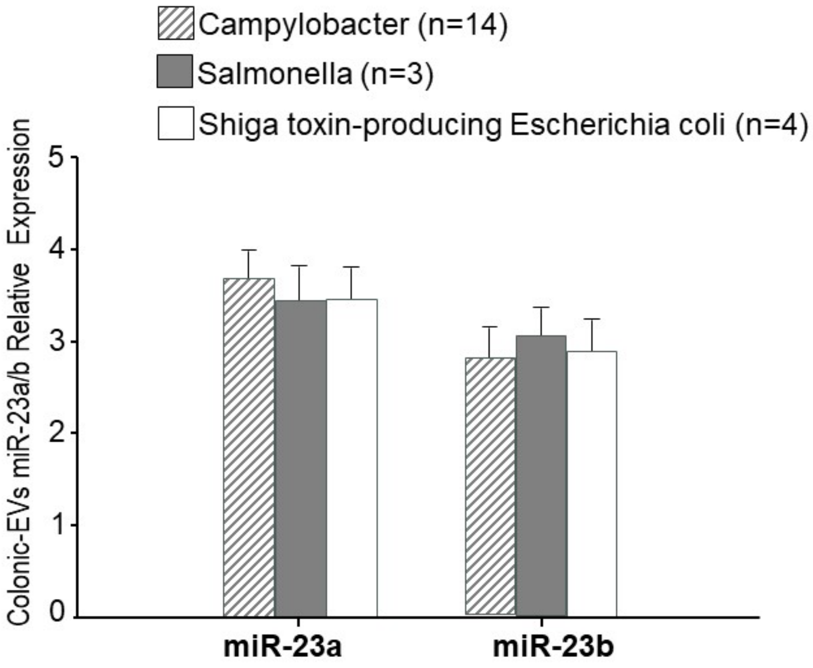

Supplementary Figure 9. miR-23a/b expression in colon EVs of PI-IBS-D patients by enteric pathogens. There were a total of 14 PI-IBS-D patients with Campylobacter infection history, 3 PI-IBS-D patients with Salmonella infection history, and 4 PI-IBS-D patients with Shiga toxin-producing Escherichia infection history. There is no significant difference in miR-23a/b expression these groups.

Supplementary Figure 10.

NR2B Expression in PI-IBS-D patients' Colon-EVs by Enteric Pathogen

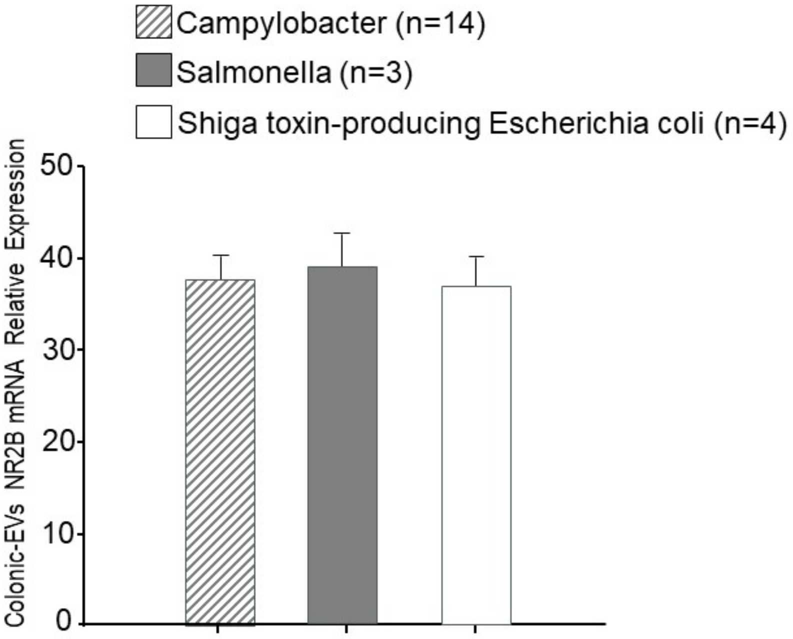

Supplementary Figure 10. NR2B expression in colon EVs of PI-IBS-D by enteric pathogens. There was a total of 14 PI-IBS-D patients with Campylobacter infection history; total 3 PI-IBS-D patients with Salmonella infection history; and total 4 PI-IBS-D patients with Shiga toxin-producing Escherichia infection history. There was no significant difference in NR2B expression between these groups.

Supplementary Figure 11

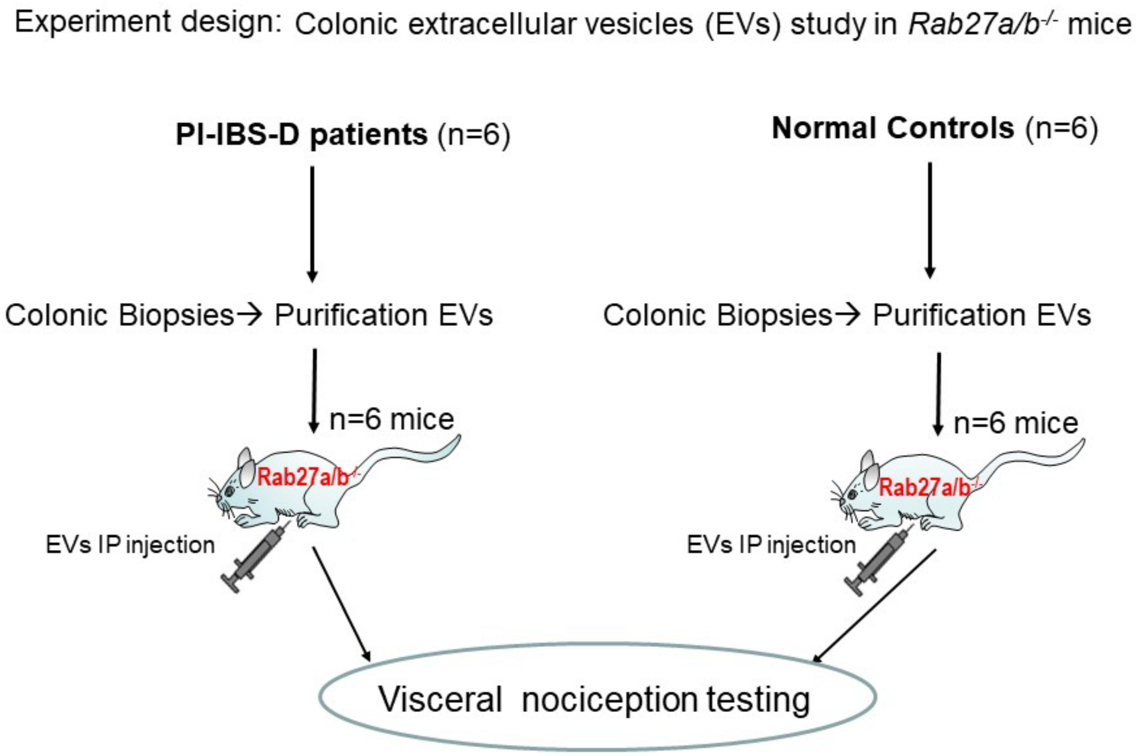

Supplemental Figure 11. Overview of experimental design: A colonic EVs study in Rab27ab$^{-/-}$ mice. To confirm the mechanistic role of EVs as internal messengers, Rab27ab$^{-/-}$ mice deficient in EV secretion were used to evaluate the EV functionality. Six Rab27a/b$^{-/-}$ mice received six individual colon EVs from patients with PI-IBS-D (left panel). Six Rab27ab$^{-/-}$ mice received six individual colon EVs from healthy controls (right panel). Visceral nociception testing (VMR to CD) was also performed.

Supplementary Figure 12

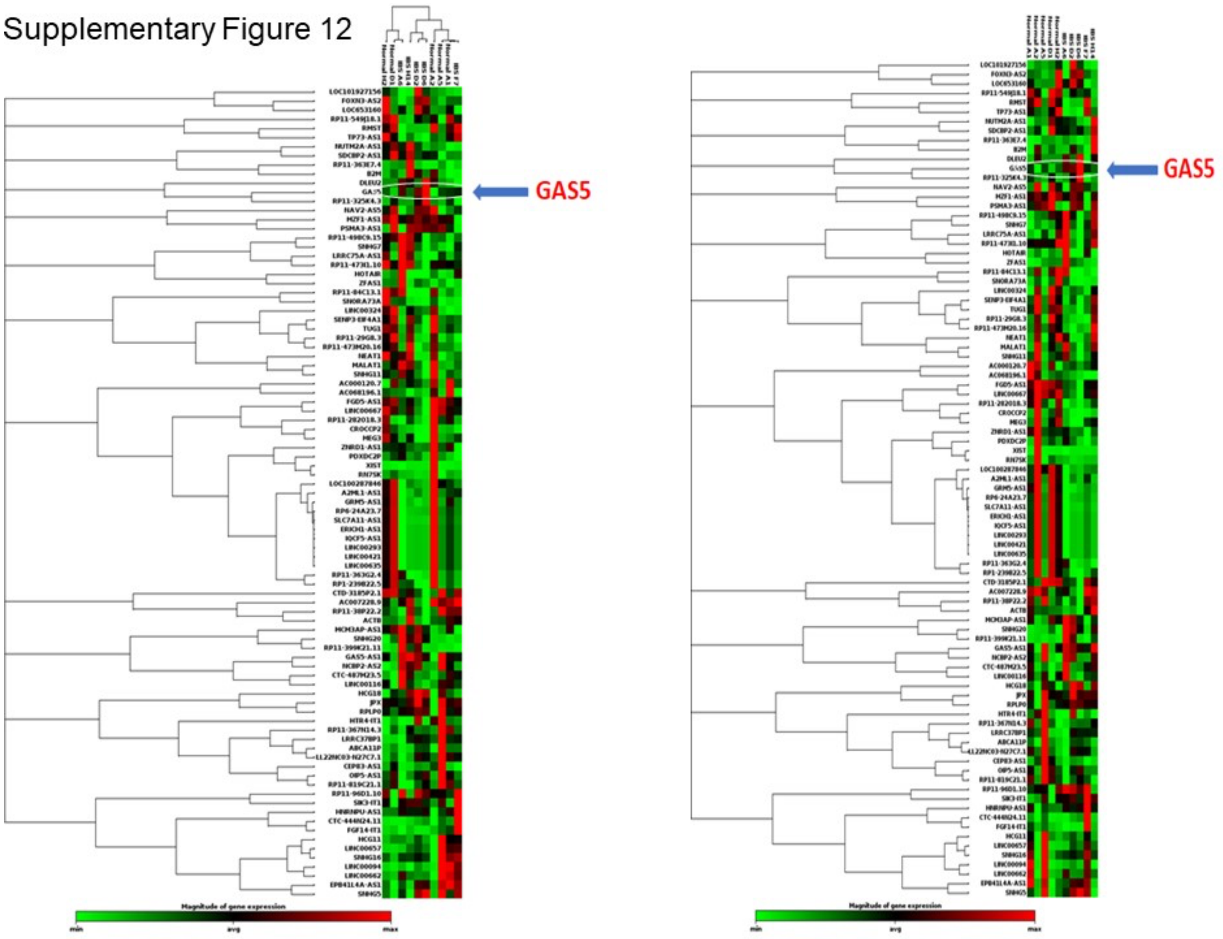

Supplementary Figure 12. Heatmap for Real-time PCR-based lncRNA profiling and super-array analysis in EVs from PI-IBS-D patients. PCR-array profiling studies using the RT² lncRNA PCR Array revealed that the expression of lncRNA GAS5 was greatly increased in colonic EVs derived from patients with PI-IBS-D.

Supplementary Figure 13

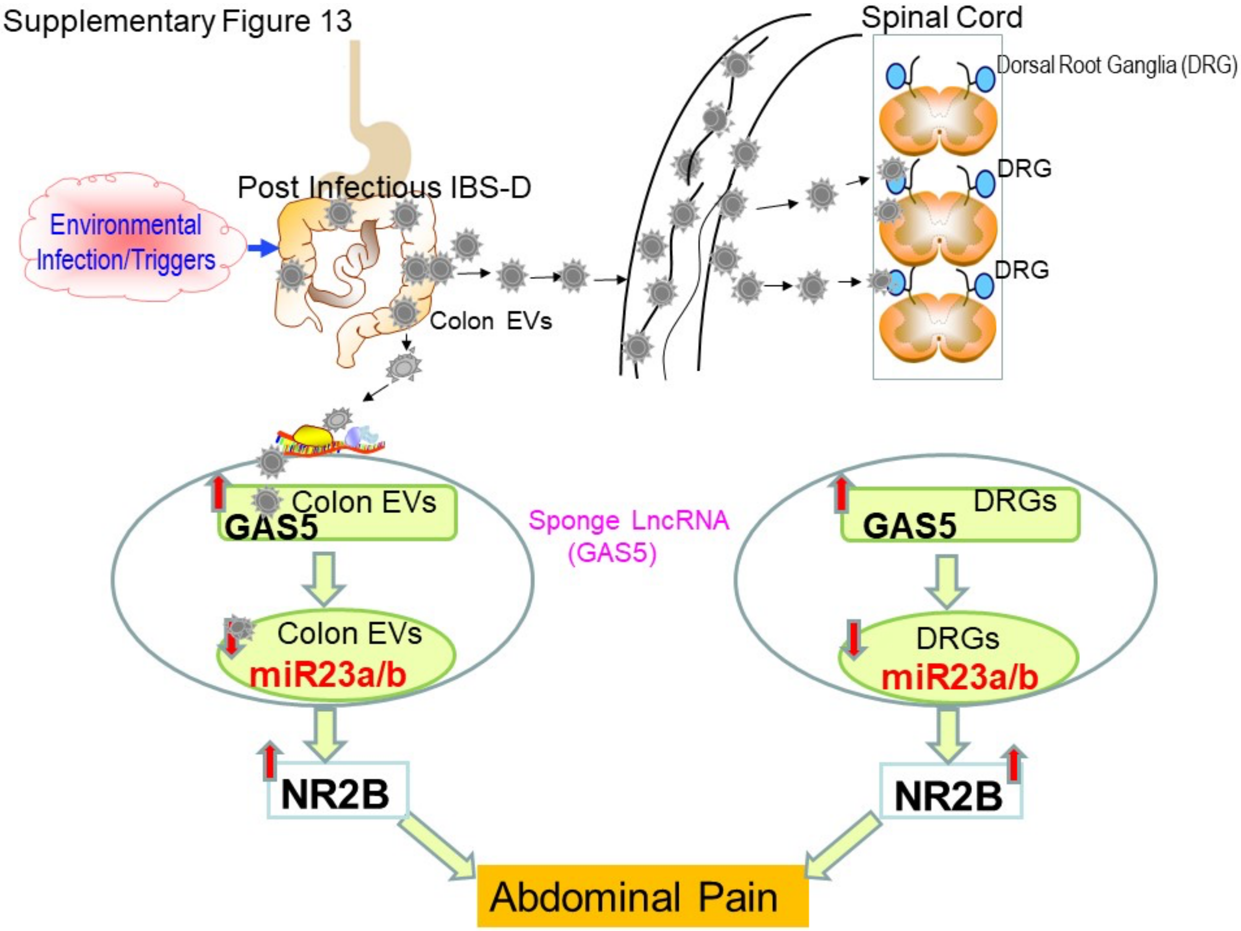

Supplementary Figure 13. The diagram illustrates that colon-EVs are released as a result of enteric infections. Colon EVs transfer information via endocytosis to areas throughout the colon (left) in addition to distant areas of the body, such as the DRGs (right). Increased levels of GAS5 in the colon EVs suppress the expression of miR-23a/b in the colon and DRGs, which increases NR2B expression, leading to increased abdominal pain.
